# Microwave-assisted nanocatalysis: A CuO NPs/rGO composite as an efficient and recyclable catalyst for the Petasis-borono–Mannich reaction†

**DOI:** 10.1039/c8ra05203d

**Published:** 2018-08-29

**Authors:** Anshu Dandia, Sarika Bansal, Ruchi Sharma, Kuldeep S. Rathore, Vijay Parewa

**Affiliations:** Centre of Advanced Studies, Department of Chemistry, University of Rajasthan Jaipur India dranshudandia@yahoo.co.in parewavijay.parewa@gmail.com; Department of Physics, Arya College of Engineering and IT Jaipur India kuldeep_ssr@yahoo.com

## Abstract

A CuO NP decorated reduced graphene oxide (CuO NPs/rGO) composite was synthesized and characterized using various analytical techniques *viz.* XRD, TEM, SEM, UV-Vis, FT-IR, EDX, XPS and CV. The activity of the catalyst was probed for the Petasis-Borono–Mannich (PBM) reaction of boronic acids, salicylaldehydes, and amines under microwave irradiation (MW). The CuO NPs/rGO composite works as a catalyst as well as a susceptor and augments the overall ability of the reaction mixture to absorb MW. The synergistic effect of MW and CuO NPs/rGO resulted in an excellent outcome of the reaction as indicated by the high TOF value (3.64 × 10^−3^ mol g^−1^ min^−1^). The catalytic activity of the CuO NPs/rGO composite was about 12-fold higher under MW compared to the conventional method. The catalyst was recovered by simple filtration and recycled 8 times without significant loss in activity. This atom-economical protocol includes a much milder procedure, and a catalyst benign in nature, does not involve any tedious work-up for purification, and avoids hazardous reagents/byproducts and the target molecules were obtained in good to excellent yields.

## Abstract 2

CuO NPs/rGO composite was synthesized and characterized by various analytical techniques an used as a recyclable catalyst for Petasis-borono–Mannich reaction under microwave irradiation.

## Graphical abstract

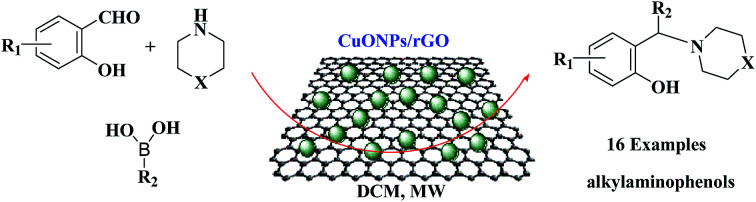

## 1. Introduction

The chemical industry depends on synthetic organic processes for the manufacture of value-added compounds for increasing productivity, discovering new leads and generating novel therapeutic agents against the vast numbers of potential drug targets.^1^ In order to implement sustainable industrial development, the use of catalysts is considered as one of the most important objectives in process design nowadays.^2^ It is certain that the development of catalysts that are low cost, have higher product selectivity, and higher reaction yields and are easily reusable is still in demand in current research scenarios. Carbon-based nanostructures have made a profound impact in many areas of science and technology due to their remarkable properties.^3^ In this direction, graphene oxide (GO) nanosheets have attracted considerable attention because of their promising applications in various fields.^4^ Ready functionalization, good water-dispersion, reusability, non-toxicity, easy availability in bulk quantities, a great deal of oxygen functionality in edge and defect sites, such as hydroxyl (–OH), carboxylic (–COOH), carbonyl (C=O), and epoxide groups (C–O–C) and high cell compatibility make GO nanosheets a vital material in the chemical sciences.^5^ GO shows great potential as a versatile catalyst for green and sustainable organic synthesis due to its high surface area and easy recyclability.^6^

Furthermore, multicomponent reactions (MCRs) have emerged as powerful routes for the synthesis of various complex molecules.^7^ MCRs in all areas of applied chemistry are very popular because they offer a wealth of products, while requiring only a minimum amount of effort. In this regard, the PBM reaction, a three component coupling reaction involving boronic acids or boronate esters, carbonyl compounds, and amines, has received a great deal of interest in organic synthesis.^8^ The PBM reaction with variation in all three components of the reaction provides a useful tool in the synthesis of structurally diverse and synthetically useful classes of compounds^9–15^ such as α-amino acids, iminocyclitols, 2*H*-chromenes, 2,5-dihydrofurans, 2-hydroxy morpholines *etc.* A large number of compounds synthesized by the PBM reaction have entered preclinical and clinical trial stages over the last few years.^16,17^ Thus, due to the proficiency of the PBM reaction, various synthetic protocols have been developed. Several catalysts and solvents have been employed to affect this transformation.^18–25^

In spite of subtle improvements of the reaction conditions, somewhere down the line these reactions still lack versatility. Most of these methods have limitations in terms of the use of expensive and hazardous chemicals, longer reaction times (12–24 h), product-diversity, tedious work-up and purification procedures, harsh reaction conditions, and unsatisfactory yields. Furthermore, the employed catalytic systems are more often than non-recoverable, thus causing the turn over number (TON) or turn over frequency (TOF) to plummet, which is significant from an industrial point of view. Therefore, further development of these catalytic systems will require advanced materials that can selectively catalyze chemical reactions with high reactivity and can be recycled through simple separation and regeneration processes.

**Scheme 1:**
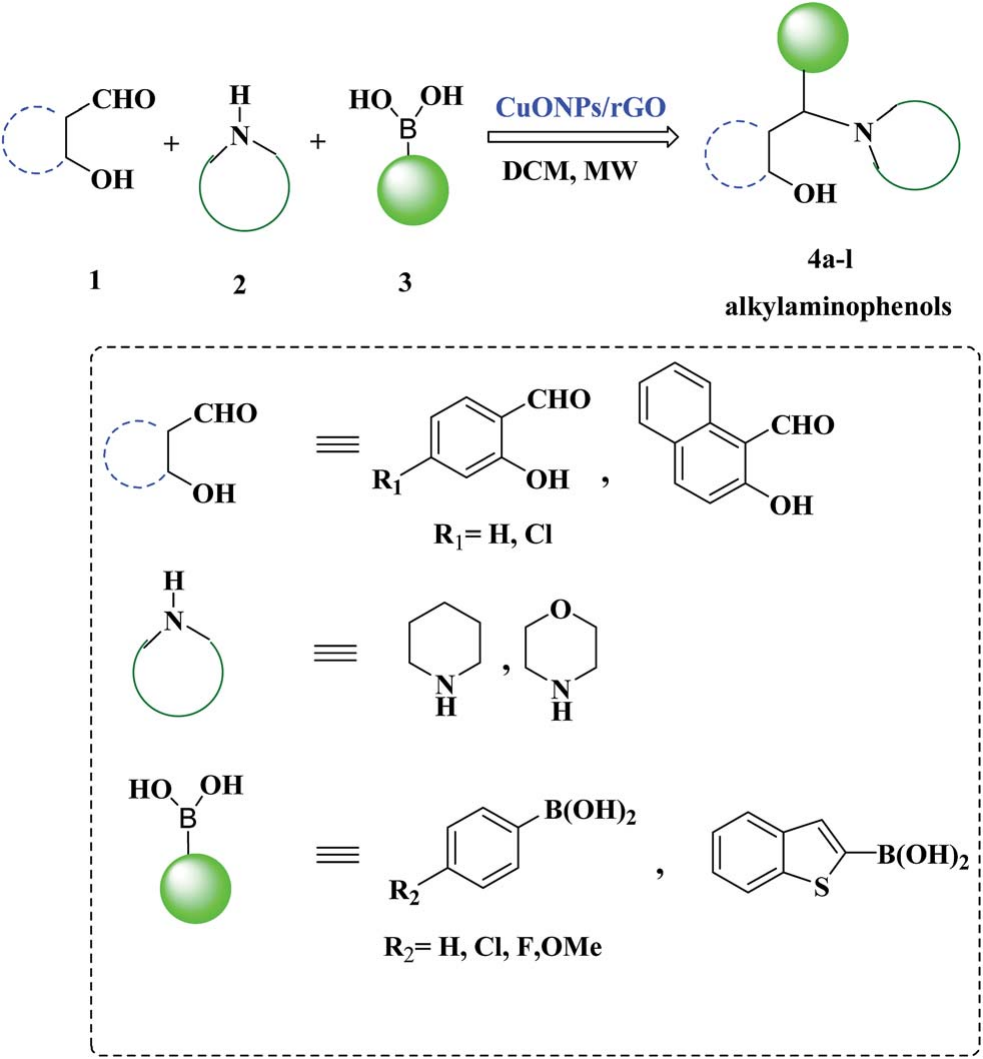
A CuO NPs/rGO composite catalyzed Petasis-Borono–Mannich (PBM) reaction.

Hence, taking into account the rationale behind the development of versatile and sustainable methodology and our interest in the exploration of expeditious synthesis of nanomaterials and heterocyclic compounds,^26–30^ herein we report the synthesis, characterization and catalytic application of a CuO NPs–rGO composite for the PBM reaction of boronic acids, salicylaldehydes, and amines under microwave irradiation (Scheme 1).

## 2. Results and discussion

### 2.1 Synthesis and characterization of CuO NPs/rGO

**Scheme 2:**
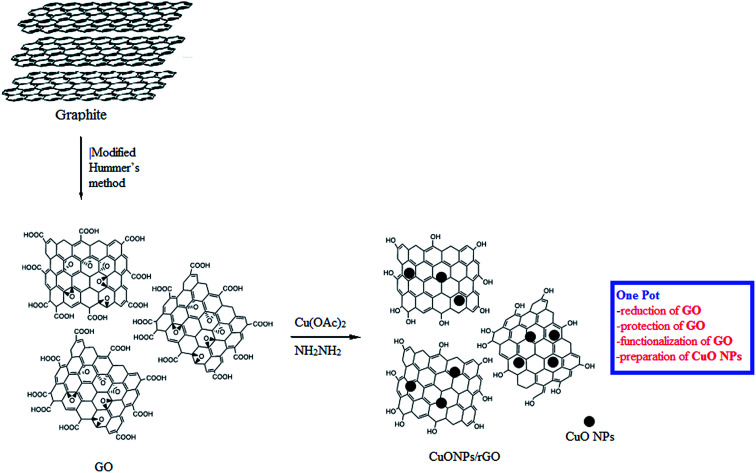
Schematic diagram for the synthesis of the CuO NPs/rGO composite.

The CuO NPs/rGO composite was synthesized by a simple, efficient and fast one-pot chemical route^31^ employing graphene oxide (GO) as a precursor of rGO, Cu(OAc)_2_ monohydrate as a precursor of CuO nanoparticles, and hydrazine hydrate as a reducing agent. This synthetic protocol involves the protection, reduction and functionalization of graphene oxide in one step (Scheme 2).

**Fig. 1:**
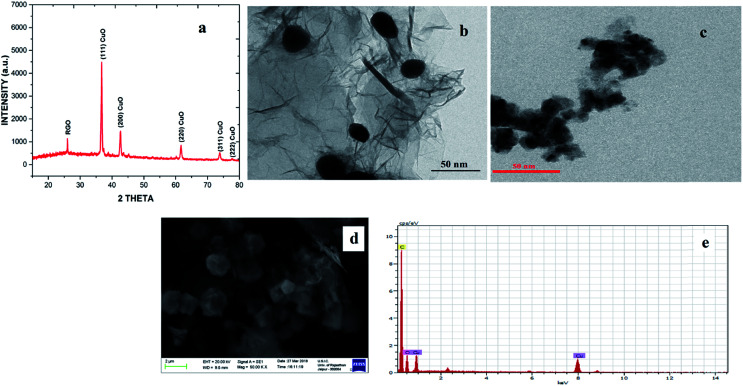
(a) XRD pattern of the CuO NPs/rGO composite, (b) TEM image of the CuO NPs/rGO composite, (c) TEM image of CuO NPs, (d) SEM image of the CuO NPs/rGO composite and (e) EDAX spectrum of the CuO NPs/rGO composite.

The CuO NPs/rGO composite was characterized by SEM, TEM, XRD, UV-Vis, FT-IR, EDX, XPS and CV. In the XRD pattern of the CuO NPs/rGO composite, diffraction peaks for crystallographic planes of the CuO NP phase and rGO are observed which indicates the successful modification of CuO NPs on the rGO sheets^32^ (Fig. 1a). These results indicate that CuO NPs were attached on the rGO surfaces and the regular interlayer structures of GO were destroyed. The average particle size of the CuO NPs was calculated to be 18 nm on the basis of the Scherrer formula. It was confirmed by TEM imaging (Fig. 1b) that the rGO sheet was modified with plenty of the CuO NPs (about 23 nm). Considering that the rGO sheet was transparent, the overlap of CuO NPs demonstrates that the particles could adsorb on both sides of the rGO. In the case of CuO NPs alone (prepared by the same method in the absence of any support), aggregation of CuO NPs led to a significantly decreased surface area (Fig. 1c).

It is clearly indicated by the SEM image that the rGO nanosheets have an irregular overlap of sheets in a layered structure due to further exfoliation during the reduction of GO to rGO. After the one-pot reduction of the copper salt and GO, CuO NPs were completely distributed on rGO sheets (Fig. 1d) and no particles were scattered out of the supports, indicating a strong interaction between the rGO support and the CuO NPs. According to EDX analysis (Fig. 1e), the CuO NPs/rGO composites contained the elements C, O, and Cu. The signals of the C and O elements originated from the support material (rGO sheets) and the signals of the O and Cu elements resulted from the decorated CuO NPs.

**Fig. 2:**
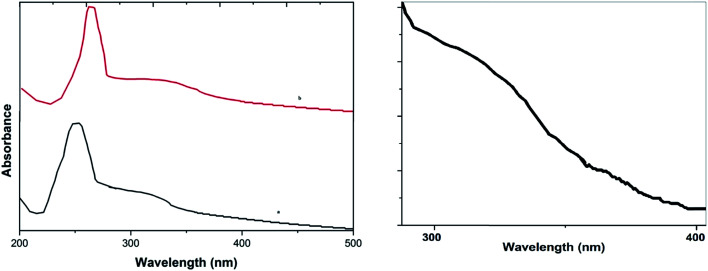
UV-Vis absorption spectra of (a) GO and the CuO NPs/rGO composite and (b) the peak for CuO in the CuO NPs/rGO composite.

UV-Vis absorption spectra (Fig. 2a) of GO, and the CuO NPs/rGO composite show that the GO dispersion exhibits a characteristic peak at 256 nm and a shoulder at 302 nm (low intensity) corresponding to π–n transitions of the aromatic C–C bonds and n–π transitions of the C–O bonds respectively (curve a). Furthermore for the CuO NPs/rGO composite (curve b), the peak at 302 nm almost disappeared and the peak at 262 nm shifted to 274 nm, which shows that GO was reduced effectively.^33^ Furthermore, a new absorption band appeared at 362 nm that could be assigned to the characteristic band of CuO,^33*b*^ indicating the presence of CuO (Fig. 2b) on the rGO sheets.

**Fig. 3:**
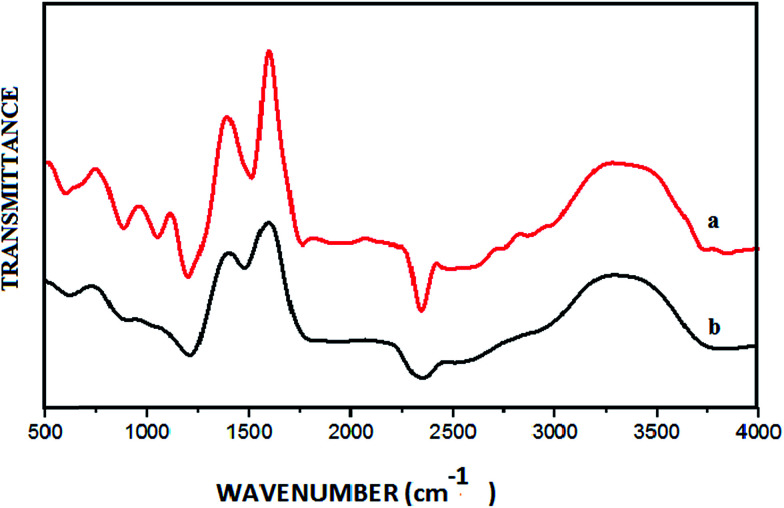
FT-IR spectra of (a) GO and (b) the CuO NPs/rGO composite.

From the FT-IR spectrum of GO (Fig. 3a), the peaks at 3378 cm^−1^ and 1720 cm^−1^ could be assigned to –OH stretching and C–O stretching vibrations respectively. The broad peak at around 1200 cm^−1^ corresponds to a C–O–C vibration. Furthermore, four absorption peaks ranging from 1400 to 1550 cm^−1^ were observed due to aromatic C=C stretching of the GO sheet. In the FT-IR spectrum of the CuO NPs/rGO composite (Fig. 3b), the peaks are relatively weak compared to those of GO. These results demonstrate the relative reduction of GO and the existence of strong interactions between the CuO NPs and the surface functionalities of the rGO sheets.^34^

**Fig. 4:**
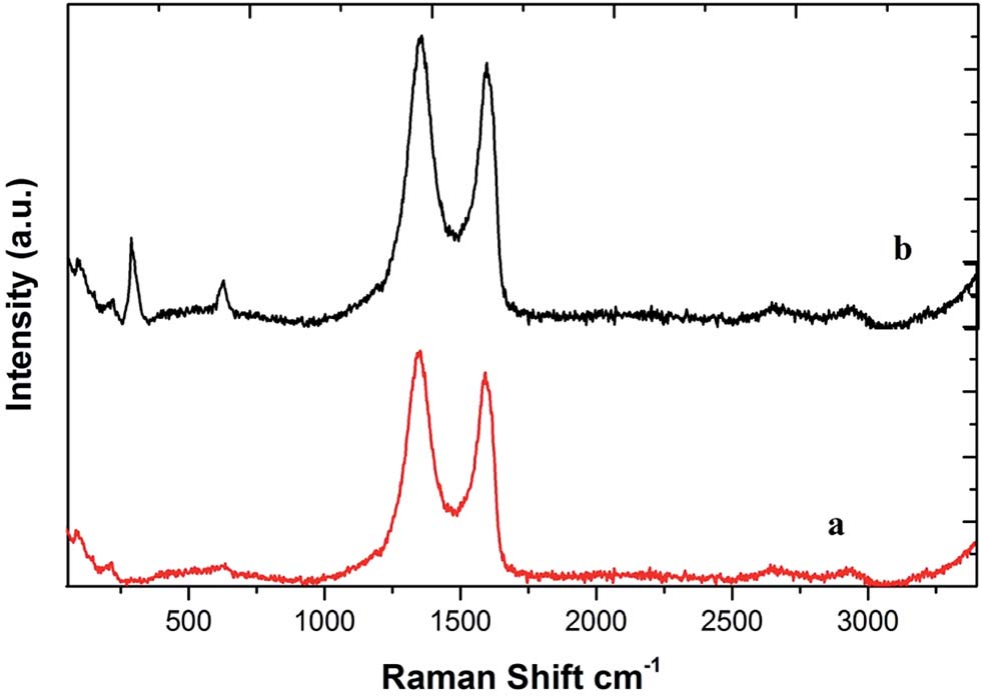
Raman spectra of GO (spectrum a) and the CuO NPs/rGO composite (spectrum b).

The Raman spectrum of GO shows a G band at around 1614 cm^−1^ and a D band at around 1356 cm^−1^ (Fig. 4; curve a). The D line arises due to the breathing mode of phonons of A_1_g symmetry near the K zone boundary; the G line originates from the in-plane vibration of sp^2^ carbon atoms and a doubly degenerate phonon mode (E_2_g symmetry) at the Brillouin zone center. Both the Raman intensities of the D and G bands slightly increased upon the adsorption of CuO NPs due to surface-enhanced Raman scattering activity (curve b).^35^ Raman bands of the CuO NPs also begin to show at 283, 323 and 647 cm^−1^ in the spectrum of the nanocomposite (curve b).^33*b*^

**Fig. 5:**
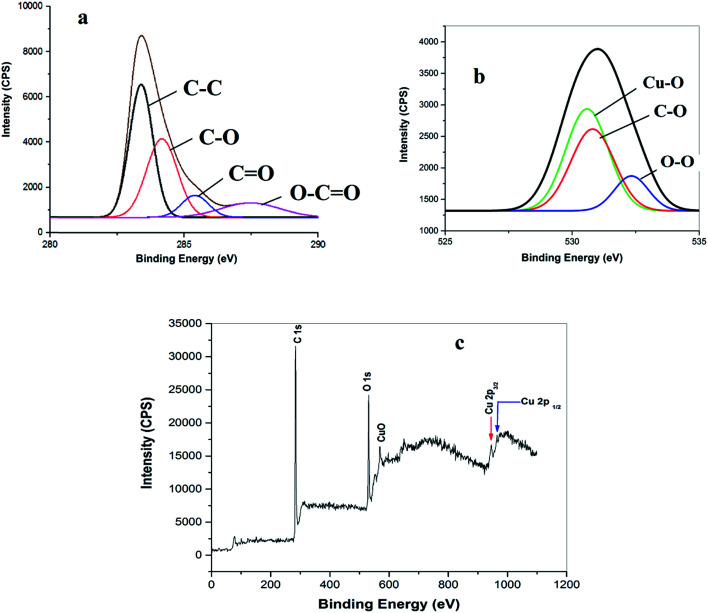
(a) C 1s, (b) O ls and (c) Cu 2p XPS survey spectra of the CuO NPs/rGO composite.

The successful preparation of the CuO NPs/rGO composite was also confirmed by X-ray photoelectron spectroscopic (XPS) measurements (Fig. 5). The deconvoluted C 1s spectrum shows four peaks at 283.8, 284.6, 285.7 and 286.5 eV which correspond to the C=C/C–C, C–O, C (epoxy)/C=O and O–C=O functionalities respectively (Fig. 5a).^36^ The O 1s XPS spectrum of the rGO–CuO nanocomposite shows peaks at 528.46, 531.45, and 532.8 eV from Cu–O, O–O and OH, respectively (Fig. 5b).^36^ Furthermore, the appearance of two signals at 944.3 eV and 965.1 eV due to Cu 2p_1/2_ and Cu 2p_3/2_ respectively in the Cu 2p XPS core level survey spectrum of the CuO NPs/rGO composite suggests the formation of metallic CuO NPs on the rGO nanosheets (Fig. 5c).^36^ The XPS results also confirm the composition of CuO (2%) and rGO (98%) in the catalyst.

The electrocatalytic responses of GO and the CuO NPs/rGO composite were obtained at pH 12.0. It was observed from the CV results that the response of GO was weak while the CuO NPs/rGO composite showed a remarkable current peak of about 5.87 μA intensity at −0.43 V which confirmed the presence of CuO in the CuO NPs/rGO composite (see ESI, Fig. S1†).

### 2.2 Catalytic activity of the CuO NPs/rGO composite

To examine the catalytic activity of the CuO NPs/rGO composite, morpholine 1 (1 mmol), phenylboronic acid 2 (1 mmol), and salicylaldehyde 3 (1 mmol) were employed as reactants for a model reaction in dichloromethane (DCM) under microwave irradiation. Initially, the mixture was treated with microwave irradiation (400 W) at 70 °C for 40 min without using any catalyst. Under these conditions the reaction did not proceed smoothly. These results encouraged us to optimize the reaction conditions. In the subsequent study, different catalysts were examined and the results are summarized in Table 1.

**Table 1:** Comparison of the CuO NPs/rGO composite with other catalytic systems for the PBM reaction to afford 4a^a^

S. no.	Catalyst	Time	Yield^b^ (%)	TOF (×10^−3^ mol g^−1^ min^−1^)
1	—	40 min	26	—
2	CuNO_3_ (10 wt%)	25 min	35	0.383
3	Cu(OAc)_2_ (10 wt%)	25 min	50	0.548
4	CuSO_4_ (10 wt%)	25 min	40	0.438
5	CuCl_2_ (10 wt%)	25 min	41	0.449
6	CuI (10 wt%)	25 min	43	0.471
7	CuO NPs (10 wt%)	15 min	71	1.30
8	GO (10 wt%)	40 min	57	0.390
9	rGO (10 wt%)	40 min	58	0.397
10	CuO NPs/rGO (10 wt%)	7 min	93	3.64
11	CuO NPs/rGO (15 wt%)	7 min	93	2.28
12	CuO NPs/rGO (5 wt%)	7 min	35	2.88

a Reactions were performed with salicylaldehyde (1 mmol), piperidine (1 mmol) and phenyl boronic acid (1 mmol) in DCM under microwave irradiation.

b Isolated yield. n.c.: not calculated.

In contrast, Cu(OAc)_2_ showed lower catalytic activity, while moderate yields were observed in the presence of various other copper salts. When CuO NPs were used as a catalyst, the reaction proceeded smoothly to afford the corresponding product in reasonable amounts (Table 1, entry 7). Further experiments were also performed to check the activity of GO and rGO sheets for this catalyzed reduction reaction and they gave good yields of the product 4a (Table 1, entries 8 and 9). In order to improve the catalyst recovery and the prevention of aggregation of NPs in the reaction mixture, CuO NPs were immobilized on a rGO support. It was found that the best results could be achieved by using the CuO NPs/rGO composite as indicated by the high TOF (3.64 × 10^−3^ mol g^−1^ min^−1^). This result is in agreement with our working hypothesis that the most surfaces of these attached CuO nanoparticles and rGO sheets are exposed to the reaction environment. Hence, higher catalytic activity was observed with the CuO NPs/rGO composite. These results show that this method is superior to the other methods in terms of yield and reaction time. The quantity of the catalyst used plays a vital role for the formation of the desired product. The results summarized in Table 1 clearly reveal that 10 wt% catalyst loading was adequate to catalyse the reaction, and excessive amount of catalyst did not increase the yield remarkably. In addition, it was also revealed that no other additive combinations such as protic or Lewis acids were at all advantageous in this method. Furthermore, we have also analyzed the results in terms of the amount of CuO loading (1%, 2%, and 3%) in the CuO NPs/rGO composite. The best results were observed when we used 10 wt% of the CuO NPs/rGO composite containing 2% CuO loading. Moreover, the composition of the CuO was characterized using an XPS technique.

In order to develop a viable approach, the model reaction was investigated under different unconventional and conventional conditions. Under MW, the catalytic activity of CuO NPs/rGO was found to be 7-fold higher than with the conventional method (Table 2).

**Table 2:** Dependency of the catalytic activity of CuO NPs/rGO on different unconventional and conventional conditions^a^

Entry	Condition	Catalyst	Temp. (°C)	Time (min)	Yield^b^ (%)	TOF (×10^−3^ mol g^−1^ min^−1^)
1	Conventional	CuO NPs/rGO (10 wt%)	70	60	65	0.297
2	Ultrasound	CuO NPs/rGO (10 wt%)	70	30	71	0.651
3	Microwave	CuO NPs/rGO (10 wt%)	50	7	68	2.66
4	Microwave	CuO NPs/rGO (10 wt%)	60	7	78	3.05
5	Microwave	CuO NPs/rGO (10 wt%)	70	7	93	3.63
6	Microwave	CuO NPs/rGO (10 wt%)	80	7	93	3.63

a Reactions were performed with salicylaldehyde (1 mmol), piperidine (1 mmol) and phenyl boronic acid (1 mmol) in DCM under microwave irradiation.

b Isolated yield.

The enhancement of the catalytic activity under MW might be due to the fact that the nanocatalyst acts as a susceptor^37^ and absorbs microwave irradiation, thus it can serve as an internal heat source for the reaction which enhances the overall capacity of the reaction mixture to absorb MW and prevents the deactivation of the nanocatalyst during the reaction. The literature supports our working hypothesis that nanomaterials are selectively heated more under MW compared to conventional heating due to differences in dielectric properties of the materials and volumetric dielectric heating under MW.^38^ During the optimization of the reaction conditions, the model reaction was also studied by varying the microwave power (300, 400, and 500 W) and the temperature. It was concluded that a 400 W power output at 70 °C was required to accomplish maximum conversion to the product. The reaction was also investigated in various solvents such as CH_3_CN, MeOH, 1,4-dioxane, THF, DMF and DCM with all other parameters kept constant and the progress of the reaction was checked through the use of TLC. The yields were found to be 46, 63, 69, 32, 42 and 92%, respectively. Hence, DCM was found to be the best solvent of choice.

These excellent preliminary results encouraged us to further explore the applicability of the CuO NPs/rGO composite for the PBM reaction (Table 3). To study the scope and limitations of this protocol, we employed a wide range of boronic acids, salicylaldehydes, and amines. In general, aryl boronic acids with electron donating groups reacted more smoothly than those possessing electron-withdrawing groups. Literature reports also reveal that boronic acids bearing electron-deficient substituents do not allow for good migration from the boron to the iminium carbon.^39^ Further extension of the scope to heteroatom containing boronic acid substrates was also investigated. With reaction times of up to 40 min, heterocyclic boronic acids afforded good yields. Moreover, the electronic environments of *o*-hydroxy aldehydes do not affect the reaction and the corresponding products were generated in good to excellent yields. In addition, different secondary amines like piperidine and morpholine were also examined, were found to be effective substrates and afforded the corresponding alkylaminophenols in high yields.

**Table 3:** CuO NPs/rGO composite catalyzed synthesis of alkylaminophenols^a^

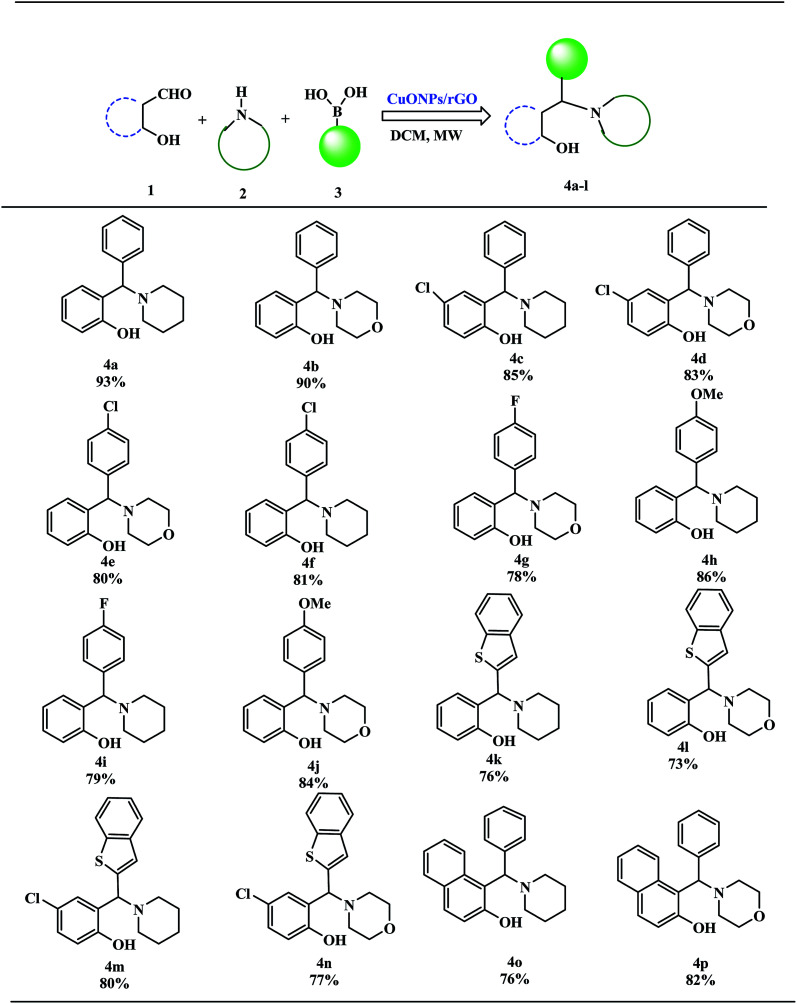

a Reaction of *o*-hydroxy aldehydes (1 mmol), secondary amines (1 mmol) and various boronic acids (1 mmol) in DCM under microwave irradiation.

**Scheme 3:**
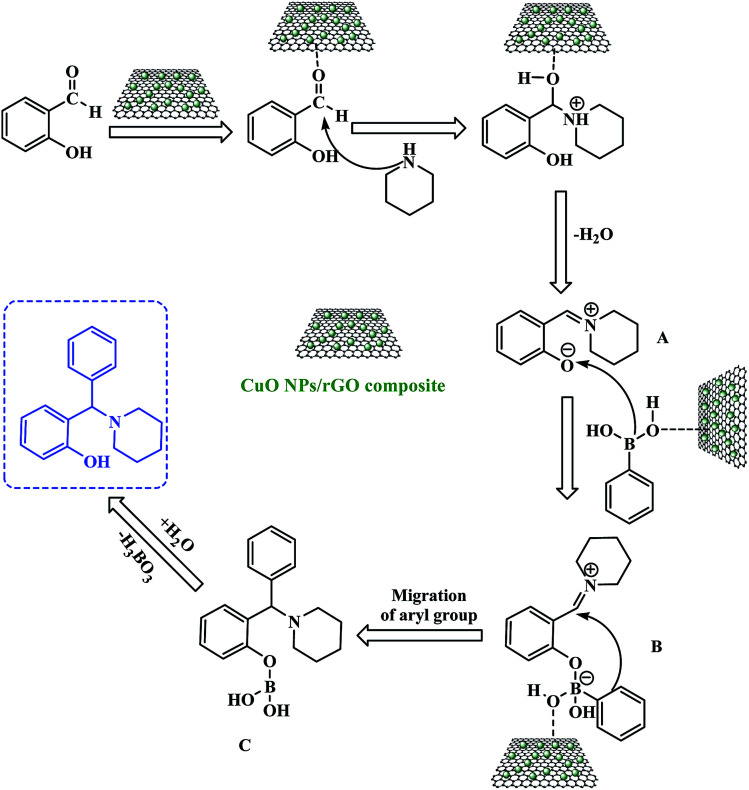
Plausible mechanism for the CuO NPs/rGO composite catalyzed PBM reaction.

A proposed reaction mechanism for this three-component PBM reaction is outlined in Scheme 3. The CuO NPs/rGO composite can serve as a Lewis acid catalyst for the reaction of salicylaldehydes and amines to give the corresponding imine (A). After that, the CuO NPs/rGO composite also facilitates the coordination between the oxygen anion of the imine (A) and the boron atom of the boronic acid leading to the formation of a tetracoordinate borate intermediate (B). Consequently, the migration of the aryl moiety of the boronic acid from the boron to the iminium carbon forms the stable intermediate (C) which upon hydrolysis gives the desired product by the loss of a H_3_BO_3_ molecule. The CuO NPs/rGO composite may enhance the rates of several of these transformations.

### 2.3 The heterogeneous nature and recyclability of the CuO NPs/rGO composite

To confirm the heterogeneous nature of the CuO NPs/rGO composite in a reaction, the model reaction was carried out again under similar reaction conditions with catalyst procured from a previous cycle. After 4 min, the catalyst was separated from the reaction mixture. The reaction was continued with the filtrate for another 30 min and the reaction conversion was monitored every 4 min. It was observed that further conversion was not observed even after 40 min. These results revealed that the reaction was occurring only due to the solid CuO NPs/rGO composite and also showed that Cu was not detached from the catalyst during the reaction. The filtrate was further analyzed by ICP-AES and there was no metallic leaching in the filtrate. This whole experiment confirms the heterogeneous nature of the presented catalytic system and the presence of strong interactions between the CuO NPs and the surface functional groups of the rGO sheets.

**Fig. 6:**
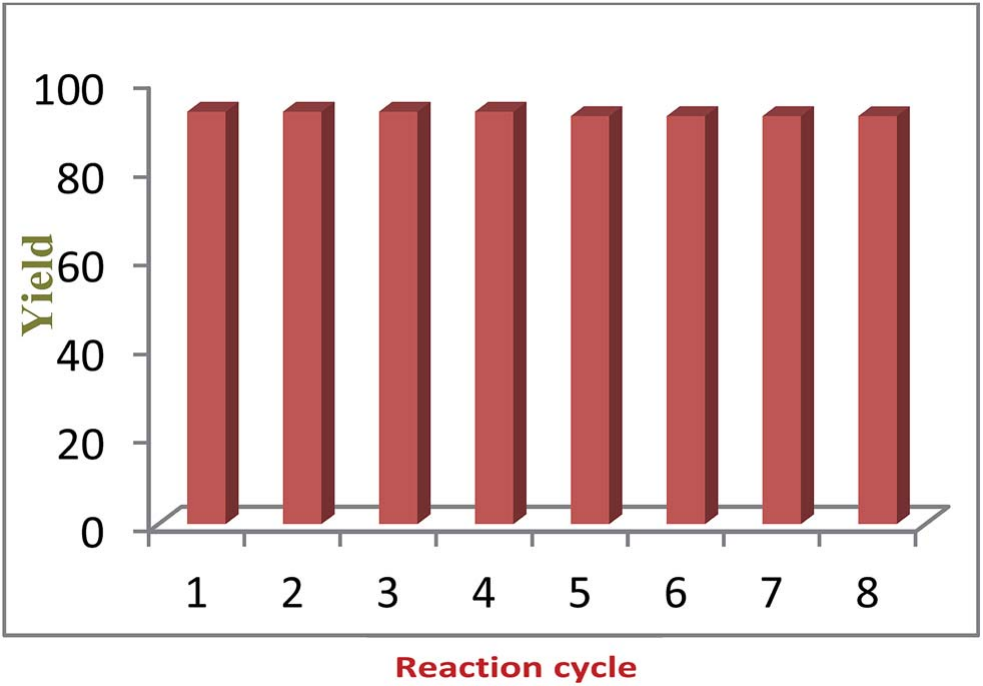
Recyclability of the CuO NPs/rGO composite.

Recycling experiments were performed by choosing the model reaction in DCM under microwave irradiation using the CuO NPs/rGO composite as a solid catalyst. When the reaction was completed, the reaction mixture was filtered and a solid precipitate was dried along with the catalyst. Then, the solid precipitate was dissolved in acetone and the catalyst was recovered by filtration. The recovered catalyst was washed with water and ethanol and reused in 8 successive reaction cycles without any significant loss in its catalytic activity (Fig. 6).

The reason that the recycling experiments were successful is that the characteristics obtained from the TEM imaging of the fresh and used catalysts were similar, which suggests retention of the structure and morphology of the CuO NPs/rGO composite after repeated use as a catalyst. According to the ICP-AES results, there was no metallic leaching in the final product. In the case of liquid products, the reaction mixtures were subjected to centrifugation in order to recover the solid catalyst.

## 3. Experimental section

General information including the instruments used is provided in the ESI.†

### 3.1 Preparation of graphene oxide (GO)

Graphene oxide (GO) was prepared using a modified Hummer’s method (see ESI†).^31^

### 3.2 Synthesis of graphene oxide sheets decorated with CuO nanoparticles

The CuO NPs/rGO composite was synthesized by a one-pot chemical route. Firstly, 200 mg of the as-prepared GO was dispersed in 250 mL deionized water and ultrasonicated for 10 min using an ultrasonic probe. The obtained dispersion was centrifuged at 10 000 rpm for 15 min to remove any unexfoliated GO. Then, Cu(OAc)_2_ monohydrate (1%, 2%, 3%) was dissolved in this dispersion and the whole material was stirred at room temperature for 30 min. After that, a 20 mL hydrazine hydrate (5 mol L^−1^) solution was added slowly and the mixture was refluxed at 90 °C under continuous stirring for 8 h. The obtained precipitate was separated by centrifugation (10 000 rpm for 15 min), washed with deionized water, and then dried under vacuum.

### 3.3 General procedure for the synthesis of alkylaminophenols 4 by the PBM reaction

*o*-Hydroxy aldehydes (1 mmol), secondary amines (1 mmol), boronic acids (1 mmol) and 10 wt% of the CuO NPs/rGO composite in DCM (15 mL) were introduced into a 50 mL round-bottom flask. The flask was placed in a microwave cavity and the reaction mixture was irradiated at 70 °C for an appropriate time at 400 W. When the reaction was completed, the resulting solid precipitate was filtered and dried along with the catalyst. Then, the solid precipitate was dissolved in acetone and the catalyst was recovered by filtration. This solution was concentrated to generate the crude product. The crude product was purified by crystallization from ethanol.

## 4. Conclusions

We have reported a very simple, convenient and green method for the selective synthesis of alkylaminophenols derivatives *via* the PBM reaction of boronic acids, salicylaldehydes, and amines catalyzed by a CuO NPs/rGO composite under microwave irradiation. The excellent results obtained from XRD, SEM, TEM, XPS, EDX, FT-IR, UV-Vis, Raman spectroscopy and cyclic voltammetry proved the feasibility and reliability of the proposed method as a facile route for the reduction of GO with *in situ* preparation and modification with CuO NPs on its surface by a one pot chemical route. Under microwave radiation, the catalytic activity of the CuO NPs/rGO composite was 12 fold higher than with the conventional method. The microwave procedure coupled with the use of the CuO NPs/rGO composite offers several advantages including cleaner reaction profiles, and highly economical, environmentally benign methodology involving shorter time durations, high yields, and simple experimental and work up procedures.

## Conflicts of interest

There are no conflicts to declare.

## Supplementary Material

RA-008-C8RA05203D-s001
