# Supplementary material for: Microwave-assisted nanocatalysis: A CuO NPs/rGO composite as an efficient and recyclable catalyst for the Petasis-borono–Mannich reaction
Source: RSC Adv. 2018 Aug 29;8(53):30280–8. doi: 10.1039/c8ra05203d (PMC9085516; doi:10.1039/c8ra05203d)
Supplement: RA-008-C8RA05203D-s001 [file RA-008-C8RA05203D-s001.pdf]

Electronic Supplementary Material (ESI) for RSC Advances.
This journal is © The Royal Society of Chemistry 2018

Microwave-assisted nano-catalysis: CuO NPs/rGO composite an efficient and recyclable catalyst for Petasis-Borono-Mannich reaction.

Anshu Dandia* [a], Sarika Bansal [a], Ruchi Sharma [a], Kuldeep S. Rathore[b], Vijay Parewa*[a]

[a]Centre of Advanced Studies, Department of Chemistry,
University of Rajasthan, Jaipur, India.

[b]Department of Physics, Arya College of Engineering and IT, Jaipur, India.

E-mail[a]: dranshudandia@yahoo.co.in

E-mail[a]: parewavijay.parewa@gmail.com

E-mail[b]: kuldeep_ssr@yahoo.com

Supporting Information

Contents: **Page No.**

1. General ……………………………………………………………………………....... 2-3
2. Heterogeneous nature and Recyclability of the catalyst ………………………………… 3-4
3. Cyclic voltammetriy (CV) of CuO NPs/rGO composite ………………………………… 5
4. Reaction System ……………………………………..…………………………………… 6
5. Characterizations of the compounds ……………………………………………………… 7-11
6. ^{1}H and ^{13}C-NMR spectra of the compounds ……………………………………....... 12-33

1. General:

All the chemicals used were of research grade (purchased from Sigma Aldrich, Acros etc.) and used without further purification. The melting points of all compounds were determined on a Toshniwal apparatus in capillary and uncorrected. ^1H and ^{13}C NMR spectra were recorded in $CDCl_3$ using TMS as an internal standard on a Bruker and JEOL NMR spectrometer at 500/400/300 and 125/100 MHz respectively. Chemical shifts are expressed in parts per million (ppm) using tetramethylsilane (TMS) as an internal standard. The following abbreviations were used to explain the multiplicities: s = singlet, d = doublet, t = triplet and m = multiplet. Mass spectrum of representative compound was recorded on Waters-Xeevo G_2S Q-Tof. X-ray diffraction (XRD) measurements for phase determination were recorded by Philips powder diffractometer (PW3040/60) with Cu K_α radiation (1.54060nm) operating in a continuous mode to collect 2θ values with a scan rate of 0.02°/min. The microwave-assisted reactions were carried out in a MAS-II microwave oven (2450 MHz, Sineo Microwave Chemistry Technology Company, Shanghai, China) with a maximum power output of 1000 W. This system is equipped with a power and temperature feedback control switch. SEM and EDX measurements were performed using a FEI Quanta 200F SEM fitted with an EDX. The size and morphology of the synthesized material was observed by transmission electron microscopy (TEM) using a JEOL 1011 at an accelerating voltage of 200kV. The UV-Vis spectra were recorded using Ocean optics USB 2000 spectrophotometer in the solution form. The Raman spectra were recorded by micro-Raman spectrometer (Jobin Yvon Horibra LABRAM-HR visible 400-1100 nm). All electrochemical experiments were performed with a CHI electrochemical analyzer, USA model no. 1230A (SR 400). IR spectra were recorded on a Shimadzu FT IR- 8400S spectrophotometer using KBr pellets.

2. Preparation of graphene oxide (GO)

Graphene oxide (GO) was prepared by the oxidation of graphite flakes according to our previously reported modified Hummer's method [50]. In this method, 3.0 g (1 wt equiv.) of graphite flakes were mixed in concentrated H_2SO_4 (69 mL). This mixture was cooled to 0 °C temperature using ice bath and put on vigorous stirring for 1 hour which resulted in the formation of black slurry. Added 1.5 g of $NaNO_3$ (0.5 wt equiv) slowly (in 25-30 min) to this slurry and whole assembly was continued for room temperature

stirring for 1 hour. Added 135 mL water to it and stirred at 80 °C for 30 minutes. Then this material was poured into 410 mL of water with constant stirring (at room temperature). After that, 20 ml of H_2O_2 was added to it gently. The resulting mixture was filtered, washed with deionized water and centrifuged (10000 rpm for 15 min twice). After discarding the supernatant, sediment was washed with deionized water, dil. HCl and ethanol respectively. During each washing, the mixture was centrifuged (10000 rpm for 15 min) and the supernatant decanted away. The obtained solid material was dried under vacuum for 20 h and referred as GO.

3. Synthesis of graphene oxide sheets decorated by CuO nanoparticles (Yield: 87%)

CuONPs/rGO composite was synthesized by one-pot chemical route. Firstly, 200 mg as prepared GO was dispersed in 250 mL deionized water and ultrasonicated for 10 min using an ultrasonic probe. The obtained dispersion was centrifuged at 10000 rpm for 15 min to remove any un-exfoliated GO. Then, $Cu(OAc)_2$ monohydrate (1%,2%,3%) was dissolved in this dispersion and the whole material was put on room temperature stirring for 30 min. After that, 20 mL hydrazine hydrate (5 mol L^{-1}) solution was added slowly to it and the mixture was shifted to refluxing at 90 °C under continuous stirring for 8 h. The obtained precipitate was separated by a centrifugation (10000 rpm for 15 min) and washed with deionized water, then dried under vacuum.

4. Heterogeneous nature and recyclability of CuO NPs/rGO composite composite

To confirm the heterogeneous nature of CuO NPs/rGO composite composite in reaction, the model reaction was carried out again under similar reaction conditions with the catalyst procured from a previous cycle. After 4 min, the catalyst was separated from the reaction mixture. The reaction was continued with filtrate for another 30 min and the reaction conversion was monitored for every 4 min. It was observed that further increment in conversion was not observed even after 40 min. These results revealed that reaction was occurring only due to the solid CuO NPs/rGO composite. It also showed that Cu was not detached from the catalyst during reaction. The filtrate was further analyzed by ICP-AES; there was no metallic leaching in filtrate. This whole experiment confirms the heterogeneous nature of present catalytic system and presence of strong interactions between CuO NPs and surface functional groups of rGO sheets.

Recycling experiments were performed by choosing the model reaction in DCM under microwave irradiation using CuO NPs/rGO composite as a solid catalyst. When reaction was completed, the reaction mixture was filtered and solid precipitate was dried along with the catalyst. Then, the solid precipitate was dissolved in acetone and catalyst was recovered by filtration. The recovered catalyst was washed with water and ethanol and reused in succeeding 8 reaction cycles without any significant loss in its catalytic activity. The reason is that the characteristics obtained from TEM of fresh and used catalysts are similar, which suggest the retention of structure and morphology of CuO NPs/rGO composite after repeated use as catalyst. According to ICP-AES results, there was no metallic leaching in the final product. In case of liquid products, the reaction mixture was subjected to centrifugation in order to recover the solid catalyst.

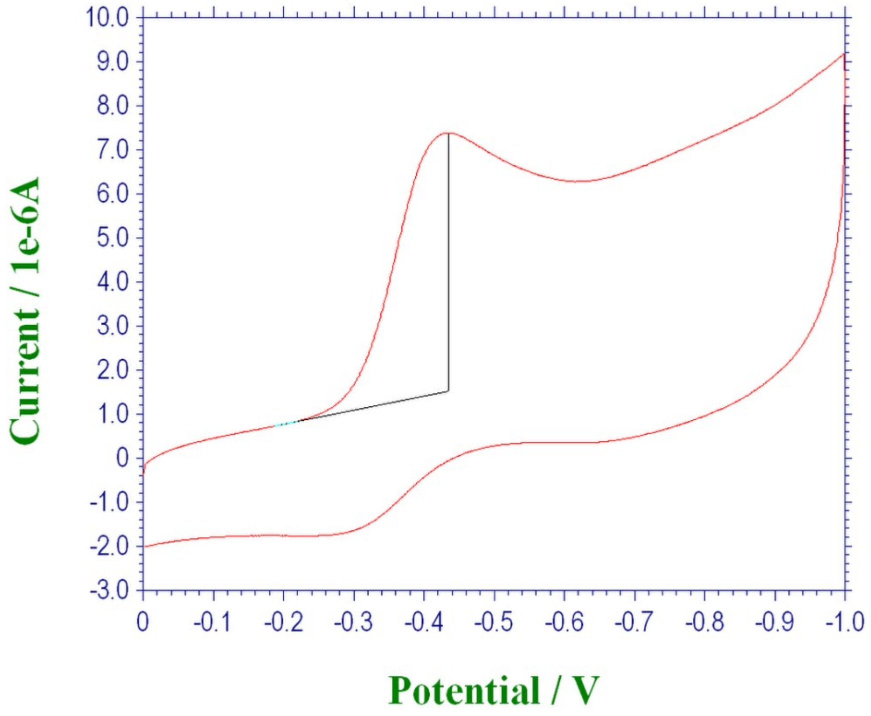

Fig. S1: Cyclic voltammetriy (CV) of CuO NPs/rGO composite at pH 12.0

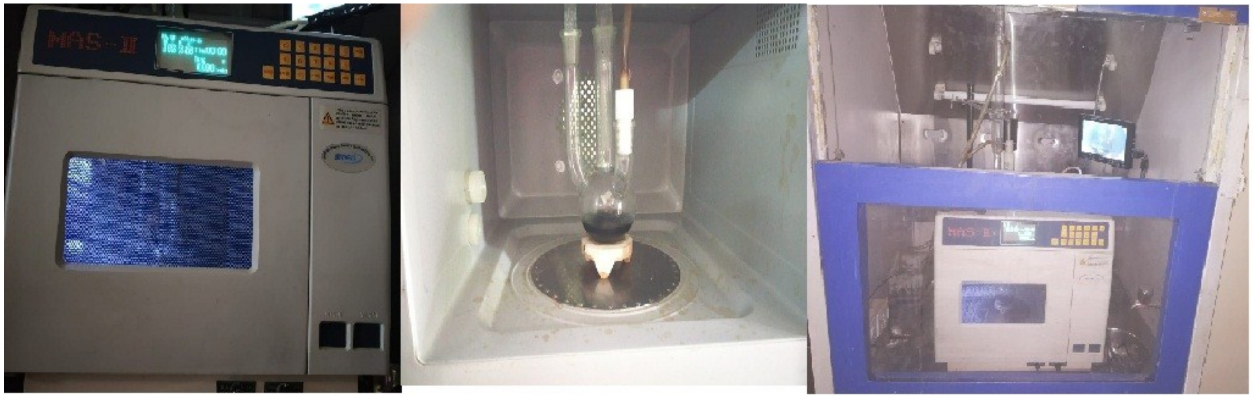

Fig. S2: Reaction System

Spectral data of synthesized compounds (4a-p):

2-(Phenyl-piperidin-1-yl-methyl)-phenol (4a)[1]

Pale yellow solid, ^{1}H NMR ($CDCl_3$, 400MHz): δ1.61-2.40(m, 10H, CH_2), 4.47(s, 1H, CH), 6.64-7.39(m, 9H, Ar-H), 11.3 (bs, 1H, OH). ^{13}C NMR ($CDCl_3$, 100MHz): δ24.20, 26.17, 52.71, 76.55, 116.9, 119.0, 125.6, 127.9, 128.4, 128.7, 129.2, 139.5, 157.2. +ESI MS (m/z): 268 $[M+H]^+$.

2-(Morpholin-4-yl-phenyl-methyl)-phenol (4b) [1]

White solid, ^{1}H NMR ($CDCl_3$, 300MHz): δ2.40-2-58(m, 4H, CH_2), 3.69-3.80(m, 4H, CH_2), 4.40(s, 1H, CH), 6.69-7.43(m, 9H, Ar-H), 11.71(bs, 1H, OH). ^{13}C NMR ($CDCl_3$, 125MHz): δ30.92, 52.25, 66.91, 117.0, 119.6, 124.8, 128.1, 128.5, 128.7, 128.9, 129.4, 139.2, 156.1. +ESI MS (m/z): 270 $[M+H]^+$.

4-Chloro-2-(phenyl-piperidin-1-yl-methyl)-phenol (4c) [2]

Pale yellow solid, ^{1}H NMR ($CDCl_3$, 300MHz): δ1.21-1.25(m, 2H, CH_2), 1.45-1.82(m, 4H, CH_2), 2.38(m, 4H, CH_2), 4.41(s, 1H, CH), 6.76-7.34(m, 8H, Ar-H), 12.67(bs, 1H, OH). ^{13}C NMR ($CDCl_3$, 125MHz): δ24.02, 26.02, 52.44, 76.08, 118.2, 123.4, 127.0, 128.1, 128.2, 128.8, 138.6, 155.9. +ESI MS (m/z): 303$[M+H]^+$.

4-Chloro-2-(morpholin-4-yl-phenyl-methyl)-phenol (4d) [4]

White solid, ^{1}H NMR ($CDCl_3$, 400MHz): δ2.41-2.45(m, 4H, CH_2), 3.73-3.75(m, 4H, CH_2), 4.34(s, 1H, CH), 6.78-7.37(m, 8H, Ar-H), 11.82(bs, 1H, OH). ^{13}C NMR ($CDCl_3$, 100MHz): δ52.25, 66.90, 76.49, 118.5, 124.0, 126.3, 128.5, 128.6, 129.1, 129.2, 138.6, 154.9. +ESI MS (m/z): 305 $[M+H]^+$

.

2-[(4-Chloro-phenyl)-morpholin-4-yl-methyl]-phenol (4e) [1]

White solid, ^{1}H NMR ($CDCl_3$, 400MHz): δ2.45-2.61(m, 4H, CH_2), 3.70-3.85(m, 4H, CH_2), 4.46(s, 1H, CH), 6.72-7.48(m, 8H, Ar-H), 11.78(bs, 1H, OH). ^{13}C NMR ($CDCl_3$, 100MHz): δ54.23, 66.91, 75.88, 117.6, 119.9, 123.9, 128.6, 129.2, 131.0, 135.6, 140.5, 156.5. +ESI MS (m/z): 305 $[M+H]^+$.

2-[(4-Chloro-phenyl)-piperidin-1-yl-methyl]-phenol (4f) [5]

Pale yellow solid, ^{1}H NMR ($CDCl_3$, 400MHz): δ1.40(m, 2H, CH_2), 1.42-1.70(m, 4H, CH_2), 2-24-2.30(m, 4H, CH_2), 4.38(s, 1H, CH), 6.60-7.30(m, 8H, Ar-H), 12.21 (bs, 1H, OH). ^{13}C NMR ($CDCl_3$, 100MHz): δ24.67, 27.80, 53.01, 75.87, 117.2, 119.3, 125.6, 126.9, 127.2, 128.9, 130.2, 133.2, 138.8, 156.5. +ESI MS (m/z): 303 $[M+H]^+$.

2-[(4-Fluoro-phenyl)-morpholin-4-yl-methyl]-phenol (4g) [5]

White solid, ^{1}H NMR ($CDCl_3$, 400MHz): δ2.30-2.50(m, 4H, CH_2), 3.55-3.95(m, 4H, CH_2), 4.48(s, 1H, CH), 6.70-7.42(m, 8H, Ar-H), 11.69(bs, 1H, OH). ^{13}C NMR ($CDCl_3$, 100MHz): δ52.55, 65.94, 76.05, 115.8, 117.5, 118.9, 124.6, 128.4, 129.2, 131.2, 139.5, 157.2. +ESI MS (m/z): 288 $[M+H]^+$.

2-[(4-Methoxy-phenyl)-piperidin-1-yl-methyl]-phenol (4h) [5]

Pale Yellow solid, ^{1}H NMR ($CDCl_3$, 400MHz): δ1.44-1.45(m, 2H, CH_2), 1.50-1.70(m, 4H, CH_2), 2.40-2.453.67 (m, 4H, CH_2), 3.67 (s, 3H, CH_3), 4.50(s, 1H, CH), 6.75-7.41(m, 8H, Ar-H), 12.61(bs, 1H, OH). ^{13}C NMR ($CDCl_3$, 100MHz): δ24.91, 26.22, 53.42, 75.15, 114.8, 116.4, 118.7, 125.2, 128.3, 130.1, 132.2, 138.4, 159.9. +ESI MS (m/z): 299 $[M+H]^+$.

2-[(4-Fluoro-phenyl)-piperidin-1-yl-methyl]-phenol (4i) [5]

Pale yellow solid, ^{1}H NMR ($CDCl_3$, 400MHz): δ1.38-1.47(m, 2H, CH_2), 1.50-1.65(m, 4H, CH_2), 2.10-2.45(m, 4H, CH_2), 4.50(s, 1H, CH), 6.75-7.40(m, 8H, Ar-H), 12.40 (bs, 1H, OH). ^{13}C NMR ($CDCl_3$, 100MHz): δ24.30, 27.17, 52.55, 75.55, 116.9, 117.4, 119.4, 125.6, 127.5, 128.4, 129.2, 131.2, 139.5, 157.2. +ESI MS (m/z): 286 $[M+H]^+$.

2-[(4-Methoxy-phenyl)-morpholin-4-yl-methyl]-phenol (4j) [1]

White solid, ^{1}H NMR ($CDCl_3$, 400MHz): δ2.40-2.55(m, 4H, CH_2), 3.70(m, 4H, CH_2), 3.78(m, 3H, CH_3), 4.44(s, 1H, CH), 6.80-7.38(m, 8H, Ar-H), 11.70(bs, 1H, OH). ^{13}C NMR ($CDCl_3$, 100MHz): δ52.55, 65.94, 76.15, 115.8, 117.5, 118.9, 125.6, 128.8, 129.6, 130.2, 140.5, 159.2. +ESI MS (m/z): 300 $[M+H]^+$.

2-(Benzo[b]thiophen-2-yl-piperidin-1-yl-methyl)-phenol (4k)

Pale yellow solid, ^{1}H NMR ($CDCl_3$, 400MHz): δ1.48-2.56(m, 10H, CH_2), 4.84(s, 1H, CH), 6.71-7.75(m, 9H, Ar-H), 11.94(bs, 1H, OH). ^{13}C NMR ($CDCl_3$, 100MHz): δ24.19, 26.17, 52.42, 71.51, 116.9, 119.3, 122.4, 123.3, 123.5, 124.4, 124.5, 124.9, 129.0, 129.0, 139.1, 140.2, 143.3, 156.7. +ESI MS (m/z): 324 $[M+H]^+$.

2-(Benzo[b]thiophen-2-yl-morpholin-4-yl-methyl)-phenol (4l)

White solid, ^{1}H NMR ($CDCl_3$, 300MHz): δ2.53(m, 4H, CH_2), 3.69(m, 4H, CH_2), 4.68(s, 1H, CH), 6.65-7.65(m, 9H, Ar-H), 11.06(bs, 1H, OH). ^{13}C NMR ($CDCl_3$, 125MHz): δ50.99, 65.85, 71.42, 116.1, 118.7, 121.3, 122.4, 122.4, 123.4, 123.6, 128.1, 128.2, 137.9, 139.1, 141.8, 154.7. +ESI MS (m/z): 326 $[M+H]^+$.

2-(Benzo[b]thiophen-2-yl-piperidin-1-yl-methyl)-4-chloro-phenol (4m)

Creamish yellow solid, ^{1}H NMR ($CDCl_3$, 500MHz): δ1.51(m, 2H, CH_2), 1.68(m, 4H, CH_2), 2.59(m, 4H, CH_2), 2.60-2.70(m, 2H, CH_2), 4.85(s, 1H, CH), 6.85-7.80(m, 8H, Ar-H), 12.08(bs, 1H, OH). ^{13}C NMR ($CDCl_3$, 125MHz): δ23.99, 25.99, 52.27, 70.93 118.3, 122.3, 123.5, 123.7, 123.7, 124.5, 124.6, 126.2, 128.6, 128.8, 138.9, 140.1, 142.1, 155.4. +ESI MS (m/z): 359 $[M+H]^+$.

2-(Benzo[b]thiophen-2-yl-morpholin-4-yl-methyl)-4-chloro-phenol (4n)

Pale yellow solid, ^{1}H NMR ($CDCl_3$, 300MHz): δ2.53 (m, 4H, CH_2), 3.68-3.78(m, 4H, CH_2), 4.48(s, 1H, CH), 6.98-7.74(m, 8H, Ar-H), 11.25(bs, 1H, OH). ^{13}C NMR ($CDCl_3$, 125MHz): δ51.95, 66.80, 71.42, 118.5, 122.4, 123.6, 123.8, 124.2, 124.6, 124.8, 125.5, 128.9, 129.2, 138.8, 140.1, 141.9, 154.5. +ESI MS (m/z): 361 $[M+H]^+$.

1-(Phenyl-piperidin-1-yl-methyl)-naphthalen-2-ol (4o) [3]

Pale yellow solid, ^{1}H NMR ($CDCl_3$, 500MHz): δ1.73-3.37(m, 10H, CH_2), 5.13(s, 1H, CH), 7.21-7.90(m, 11H, Ar-H), 14.03(bs, 1H, OH). ^{13}C NMR ($CDCl_3$, 125MHz): δ24.18, 26.11, 54.88, 72.13, 116.2, 120.0, 121.1, 122.3, 126.3, 127.9, 128.6, 128.9, 129.3, 132.4, 139.7, 155.5. +ESI MS (m/z): 318 $[M+H]^+$.

1-(Morpholin-4-yl-phenyl-methyl)-naphthalen-2-ol (4p) [3]

Pale orange solid, ^{1}H NMR ($CDCl_3$, 500MHz): δ2.33-3.85(m, 8H, CH_2), 5.17(s, 1H, CH), 7.19-7.90(m, 11H, Ar-H), 13.16(s, 1H, OH). ^{13}C NMR ($CDCl_3$, 125MHz): δ51.88, 66.90, 72.06, 115.1, 119.8, 121.0, 122.6, 126.6, 128.2, 128.8, 128.9, 129.8, 138.6, 154.7. +ESI MS (m/z): 320 $[M+H]^+$.

Reference:-

1. R. Hosseinzadeh, Z. Lasemi, M. Oloub, M. Pooryousef, J. IRAN CHEM SOC. 14 (2017) 347-355.`

2. W. Chen, Y.K. Kang, R.G. Wilde, D. Seidel, Angew. Chem. Int. Ed. 53 (2014) 5179 -5182.

3. M.L. Deb, S.S. Dey, I. Bento, M.T. Barros, C.D. Maycock, Angew. Chem. Int. Ed. 52 (2013) 9791-9795.

4. A.R. Katritzky, A.A.A. Abdel-Fattah, D.O. Tymoshenko, S.A. Belyakov, I. Ghiviriga, P.J. Steel, J. Org. Chem. 64 (1999) 6071-6075.

5. X. Shi, D. Hebrault, M. Humora, W.F. Kiesman, H. Peng, T. Talreja, Z. Wang, Z. Xin, J. Org. Chem. 77 (2012) 1154-1160.

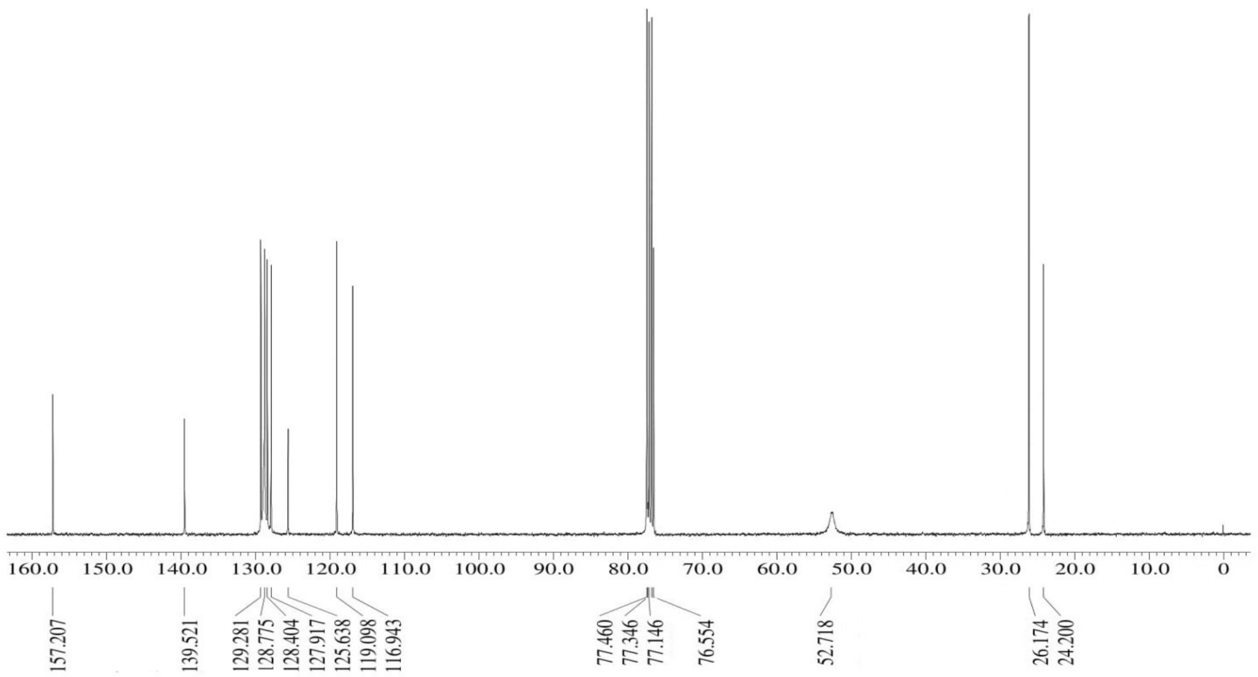

Figure 1: ^{13}C NMR spectrum of **(4a)**

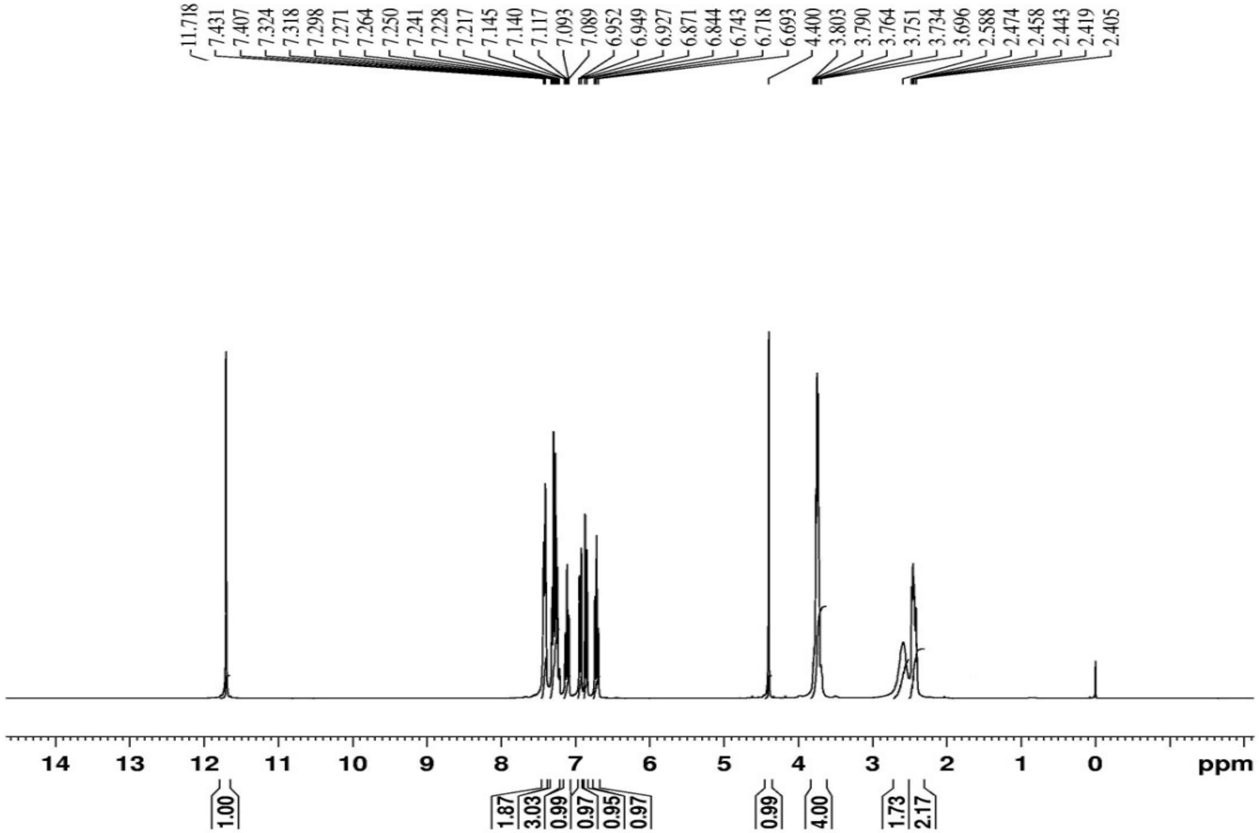

Figure 2: ^{1}H NMR spectrum of **(4b)**

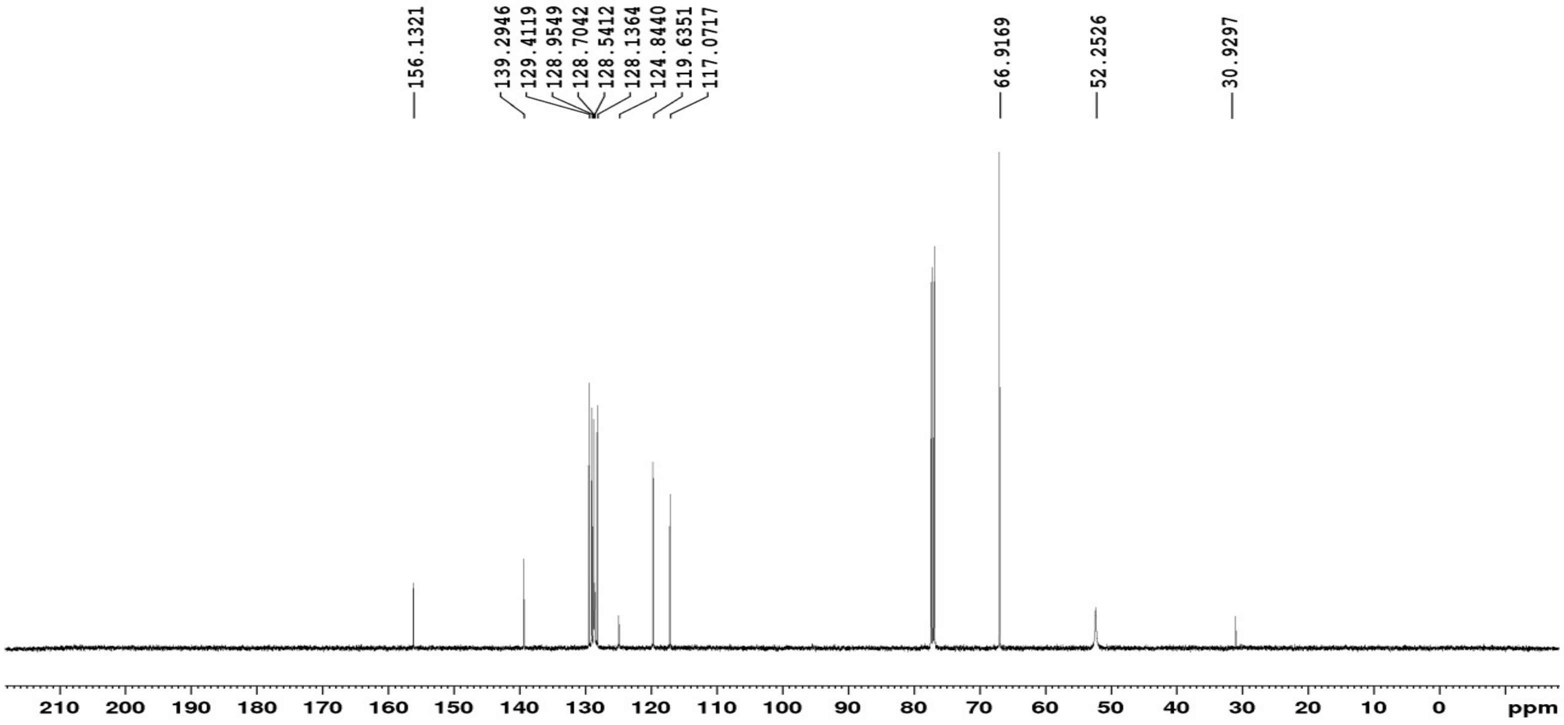

Figure 3: ^{13}C NMR spectrum of **(4b)**

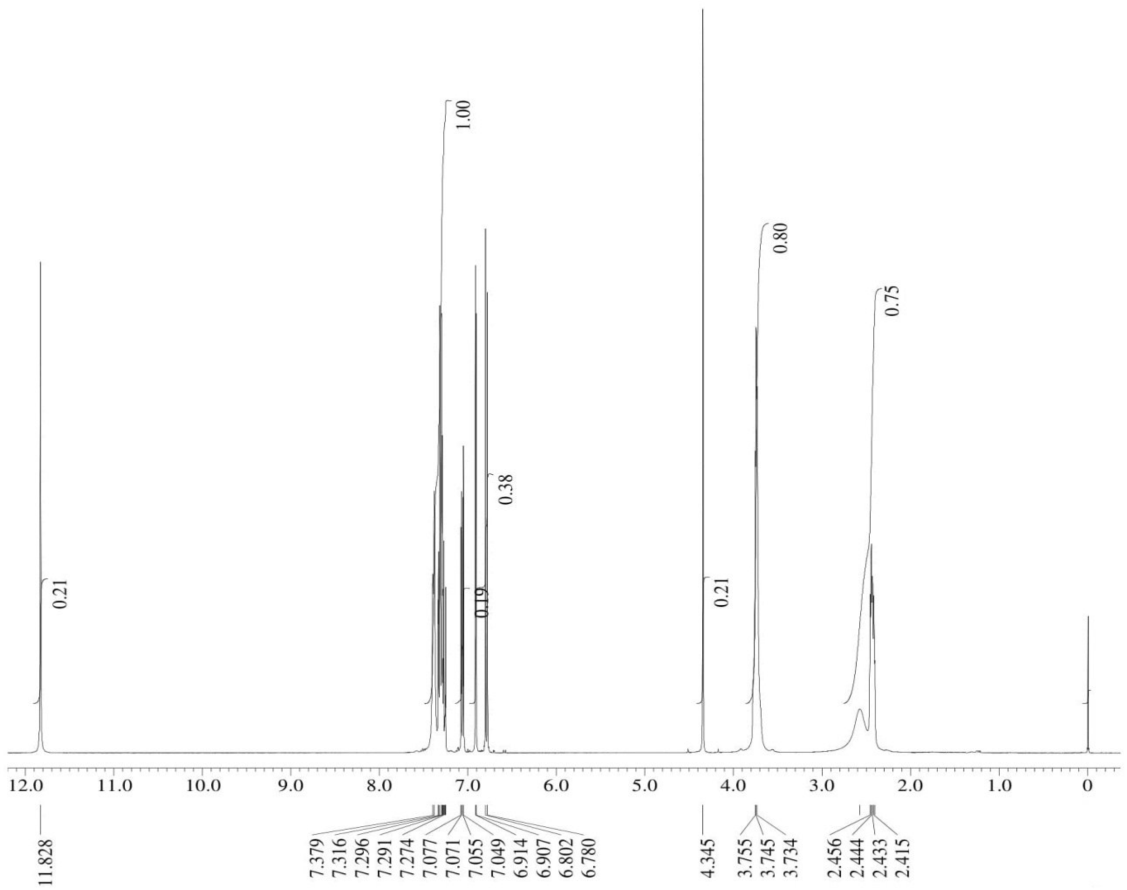

Figure 4: ^{1}H NMR spectrum of **(4d)**

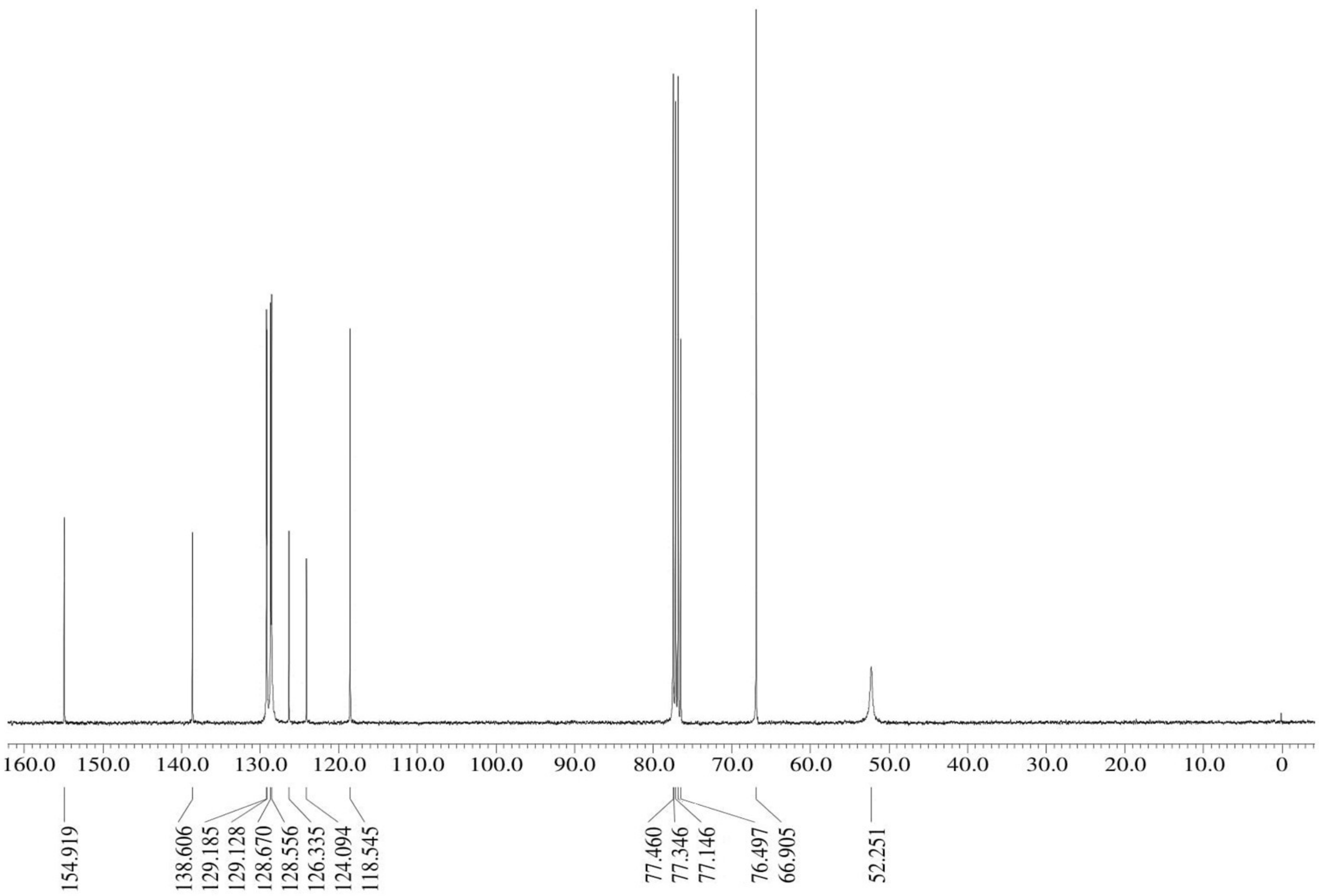

Figure 5: ^{13}C NMR spectrum of **(4d)**

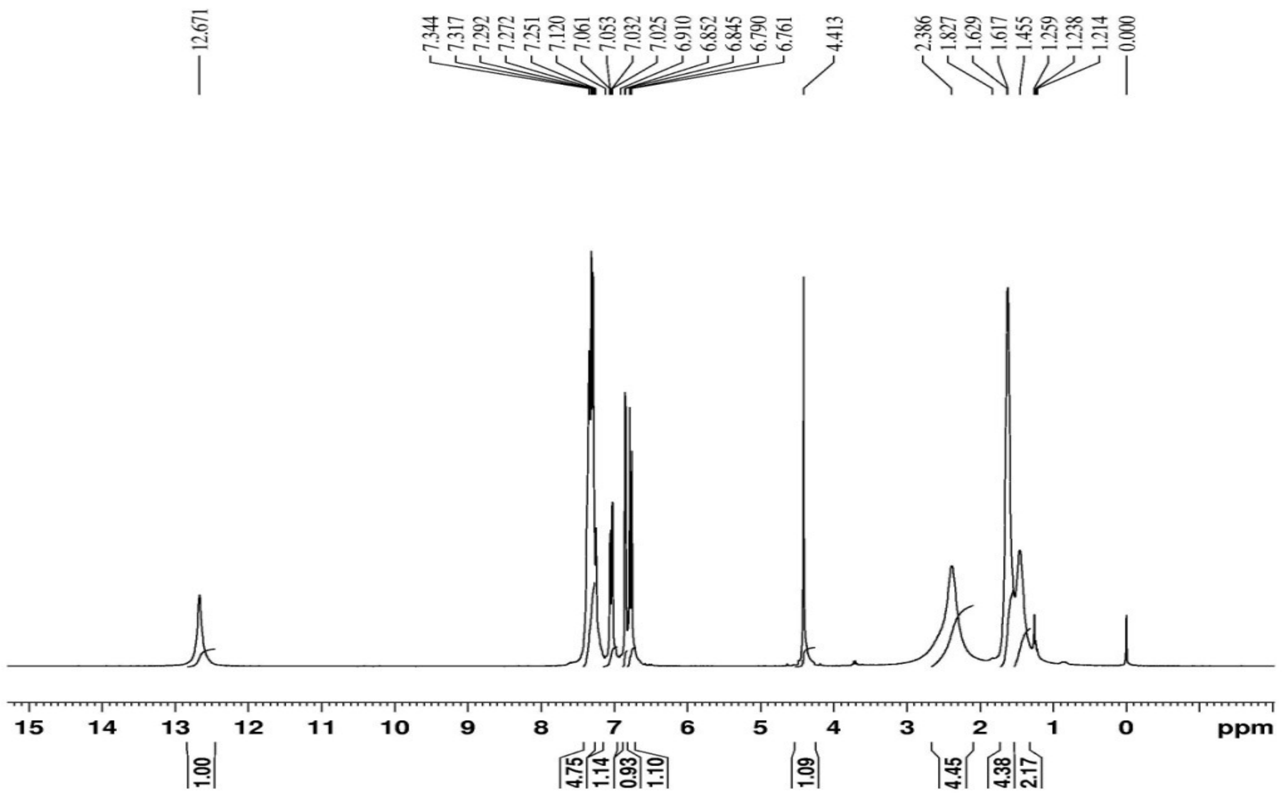

Figure 6: ^{1}H NMR spectrum of **(4c)**

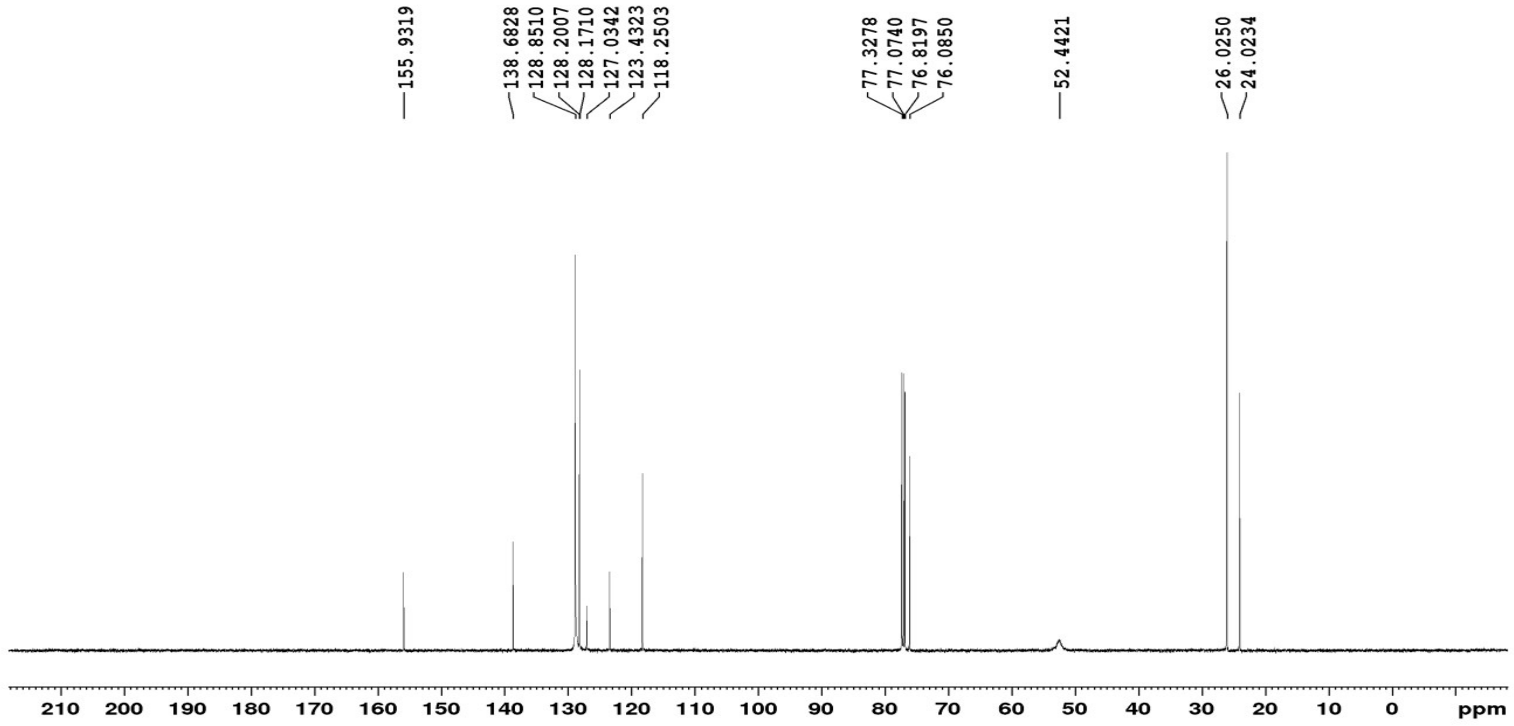

Figure 7: ^{13}C NMR spectrum of **(4c)**

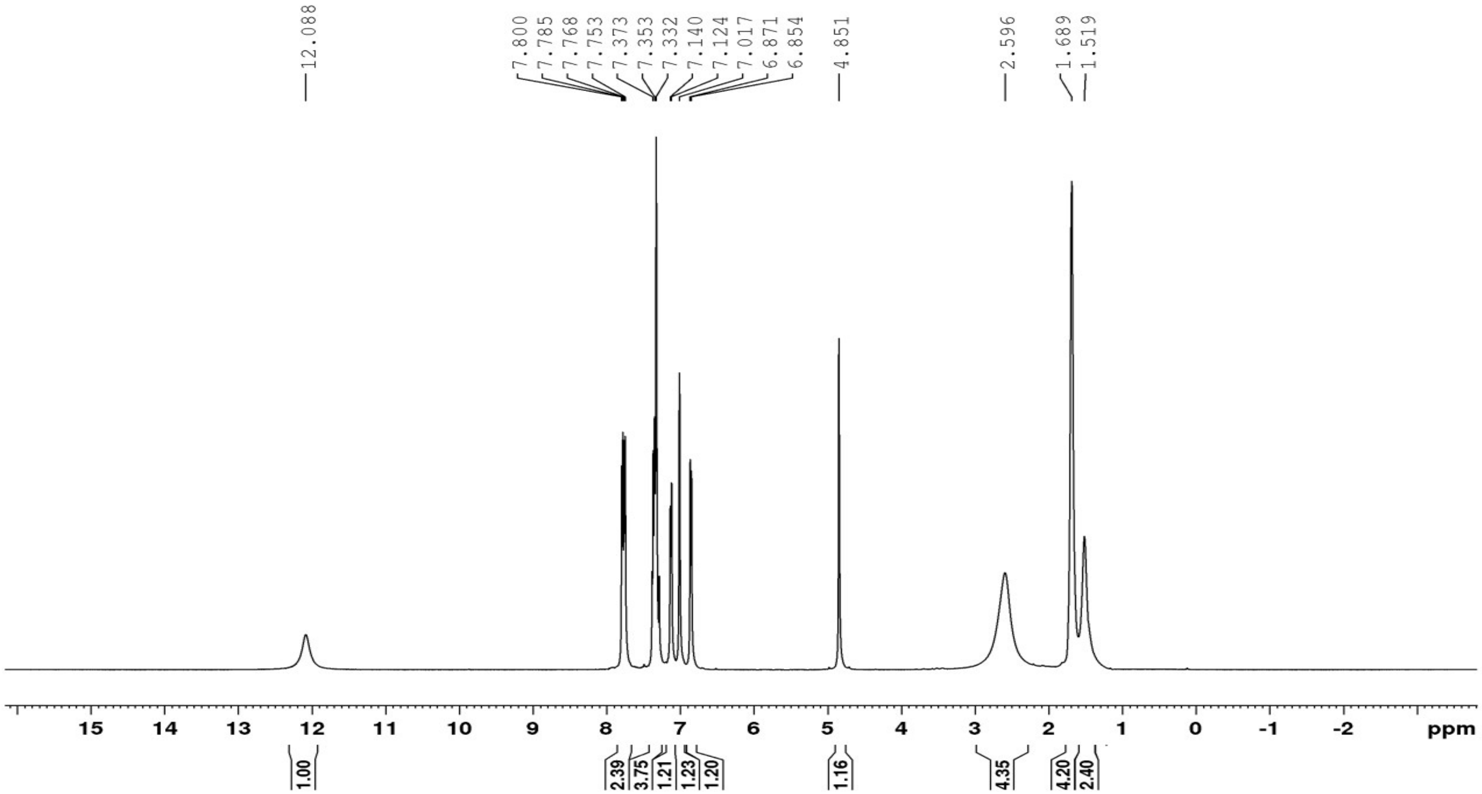

Figure 8: ^{1}H NMR spectrum of **(4m)**

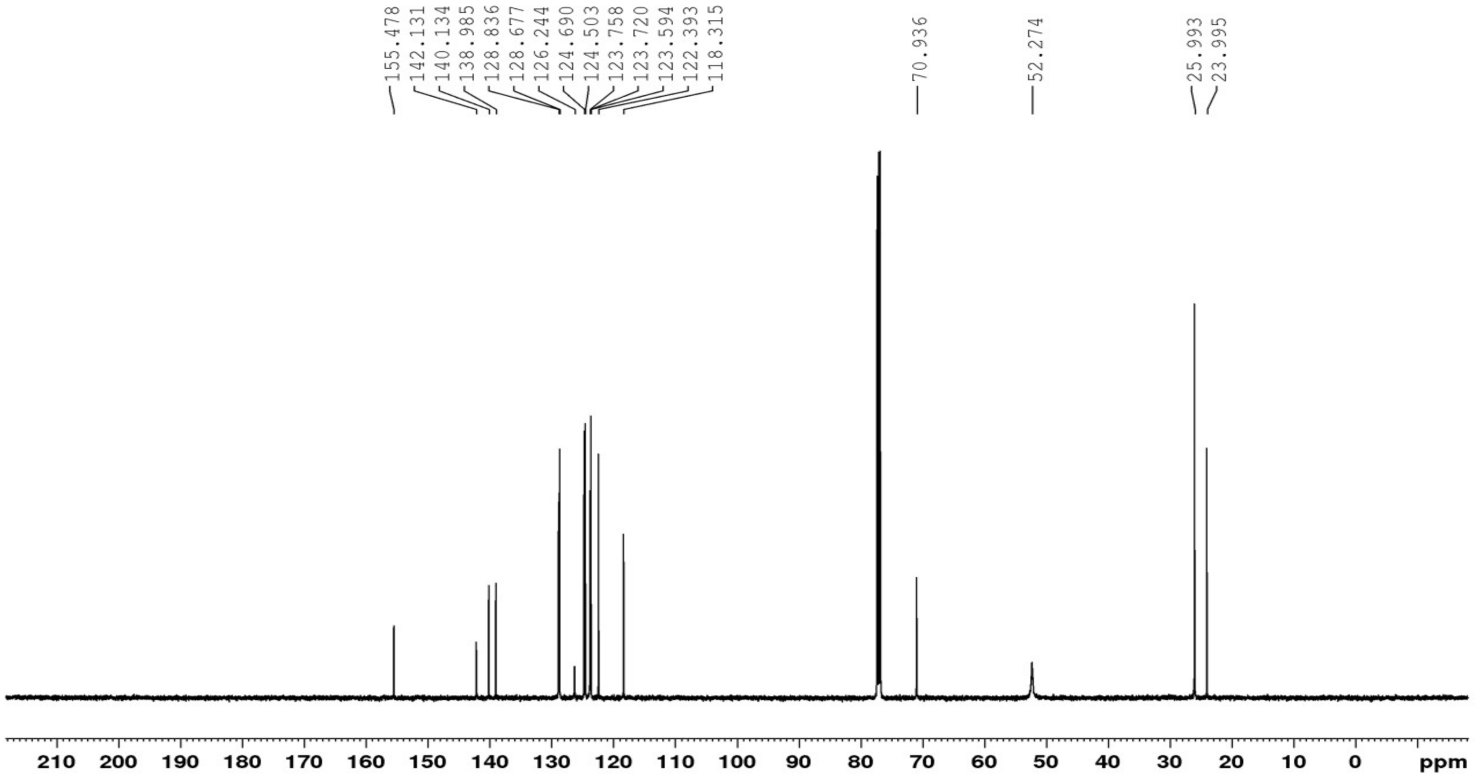

Figure 9: ^{13}C NMR spectrum of **(4m)**

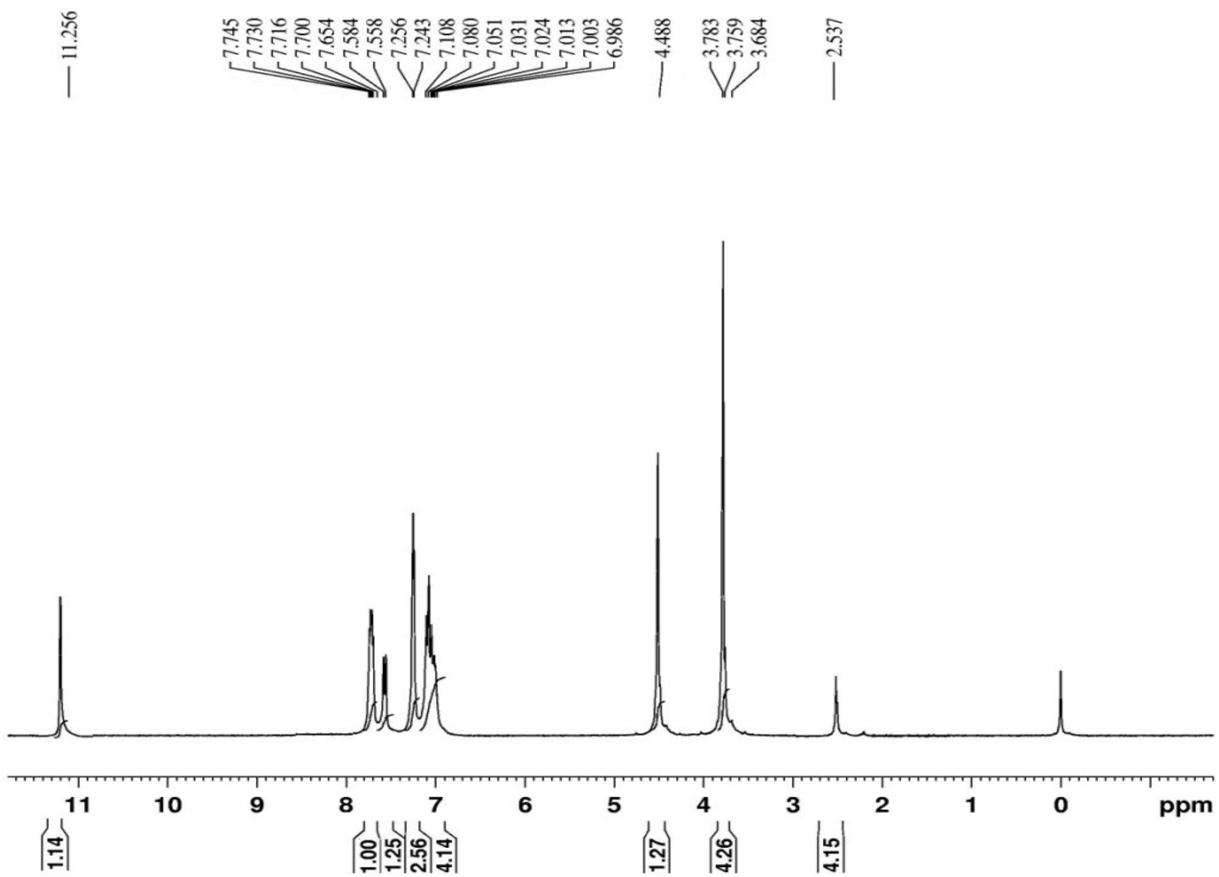

Figure 10: ^{1}H NMR spectrum of **(4n)**

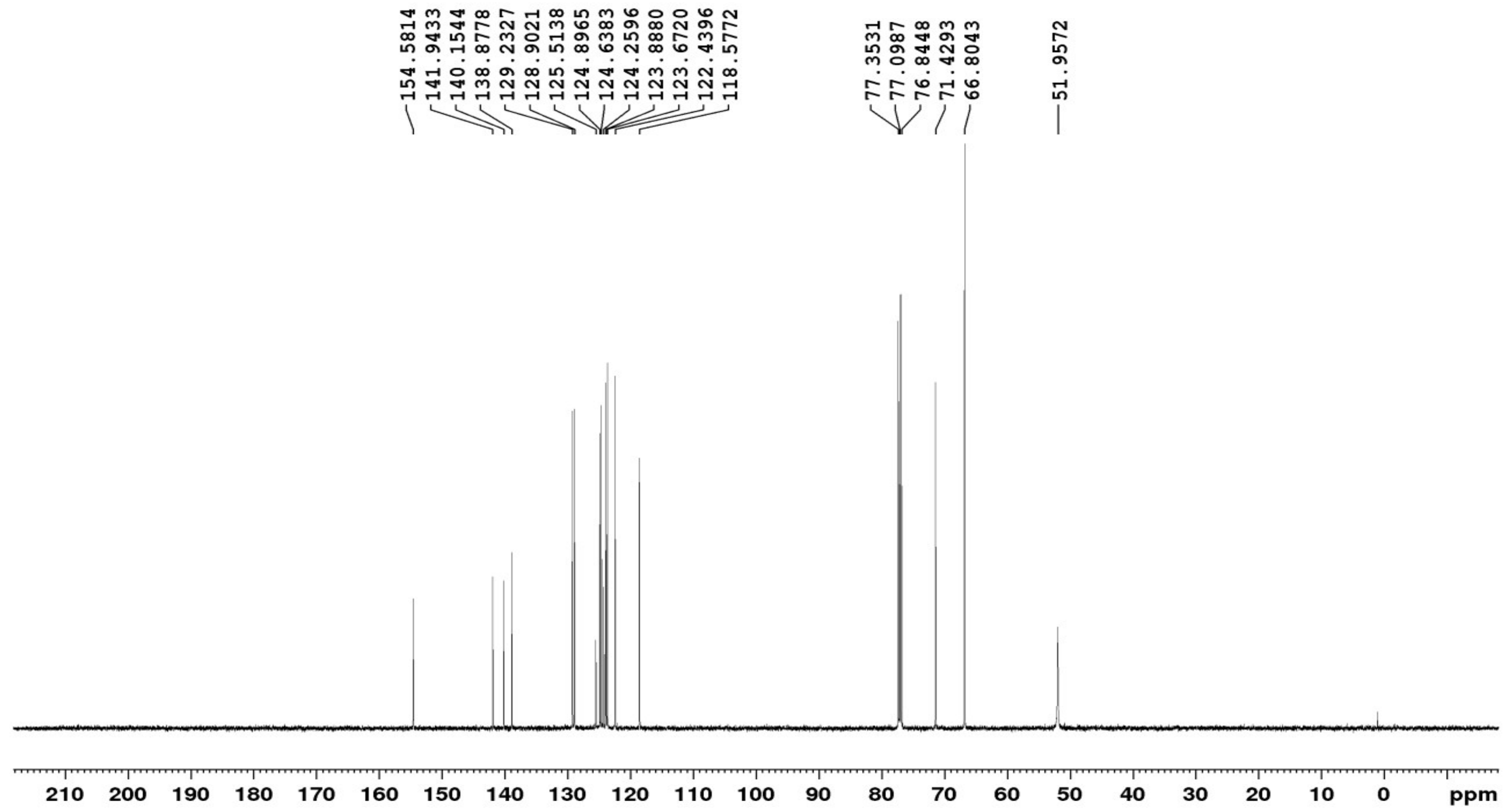

Figure 11: ^{13}C NMR spectrum of **(4n)**

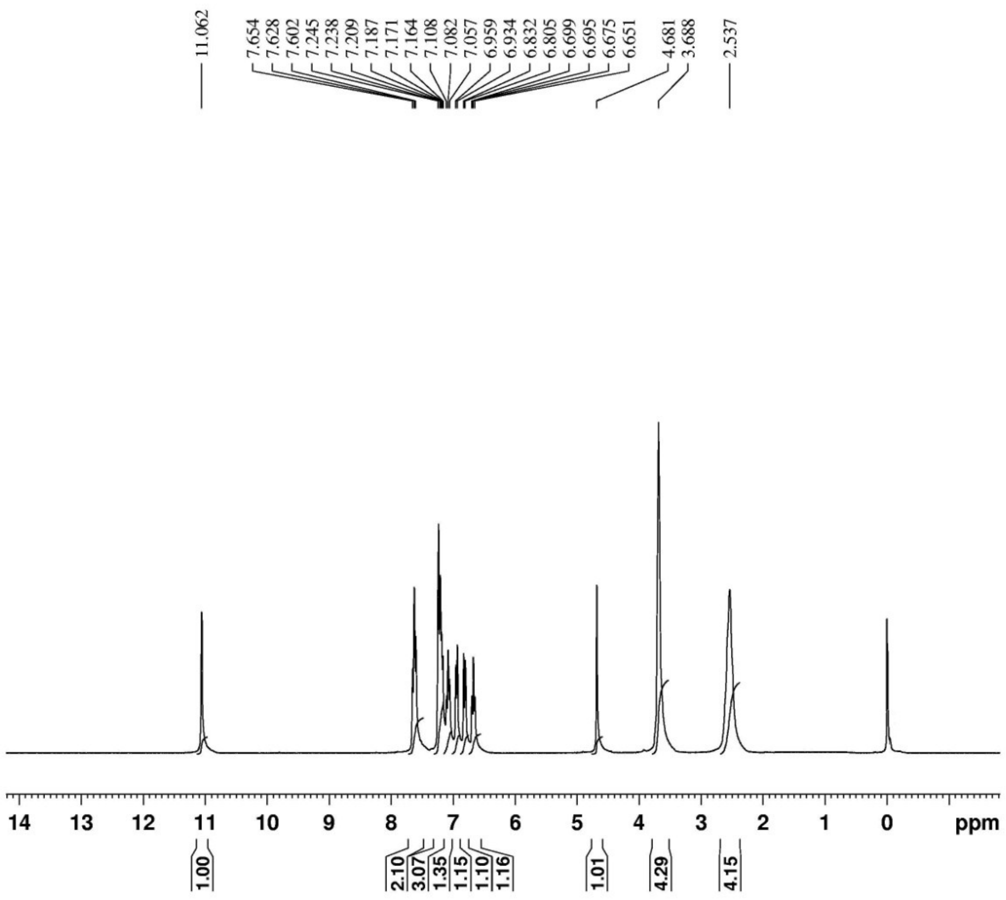

Figure 12: ^{1}H NMR spectrum of **(4l)**

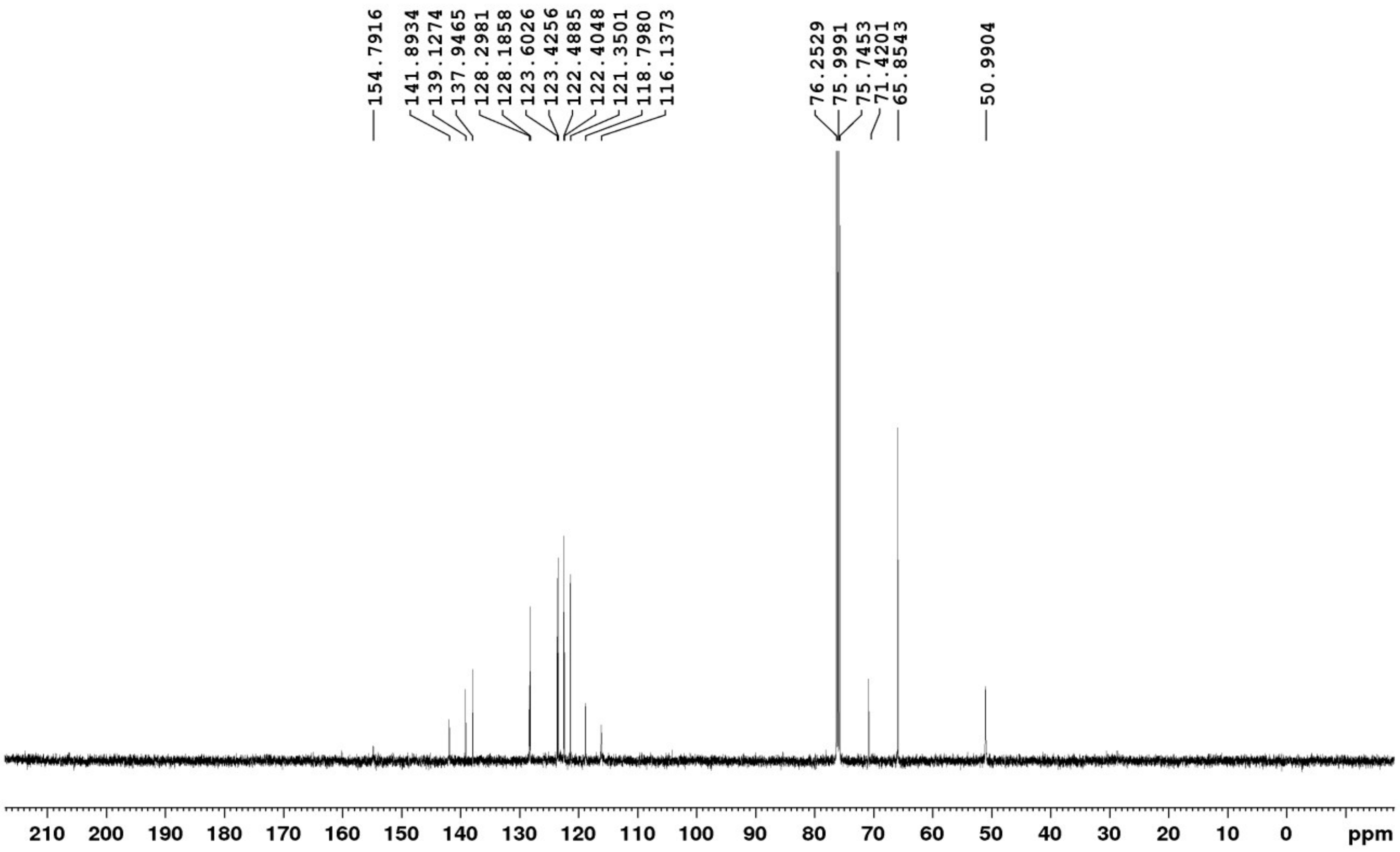

Figure 13: ^{13}C NMR spectrum of **(4l)**

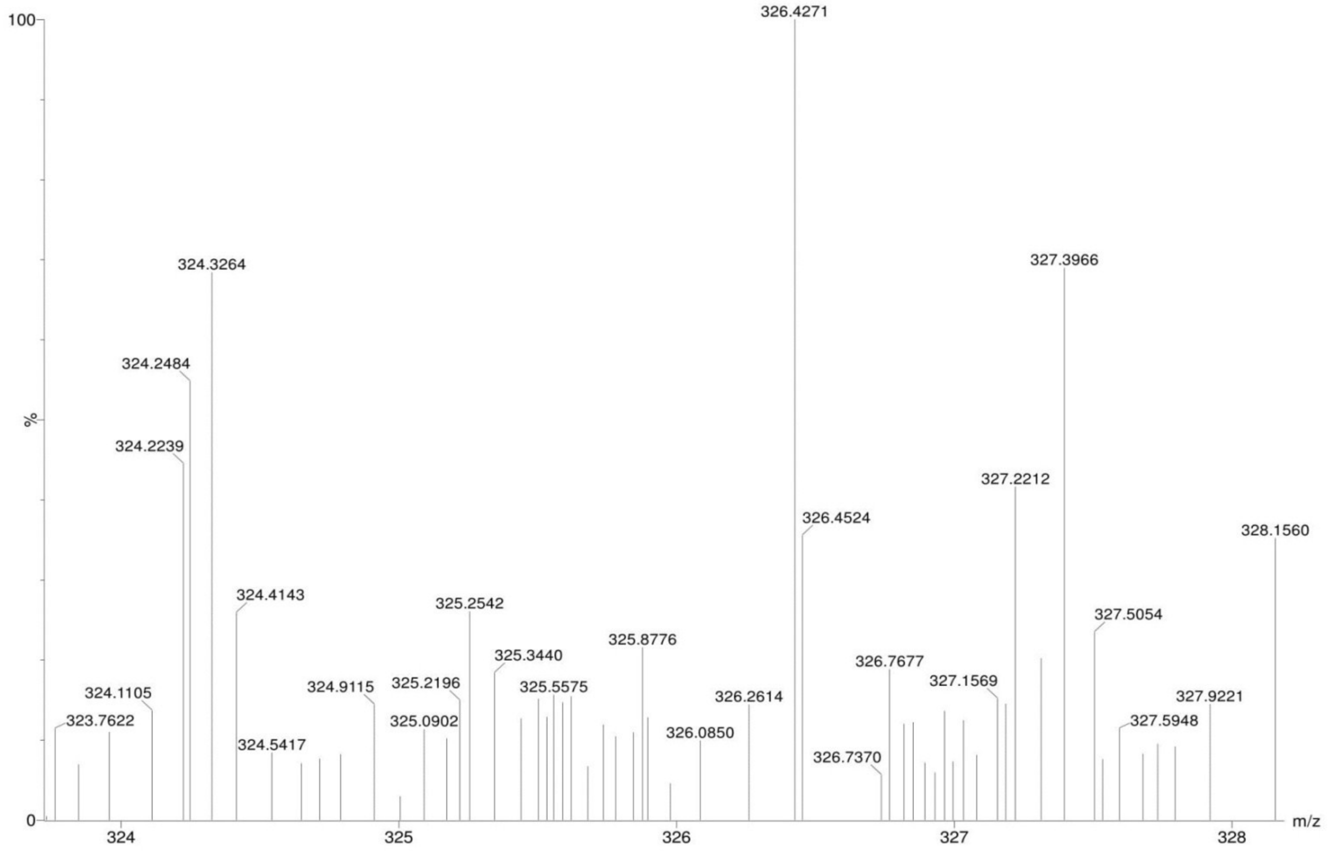

Figure 14: Mass spectrum of **(4l)**

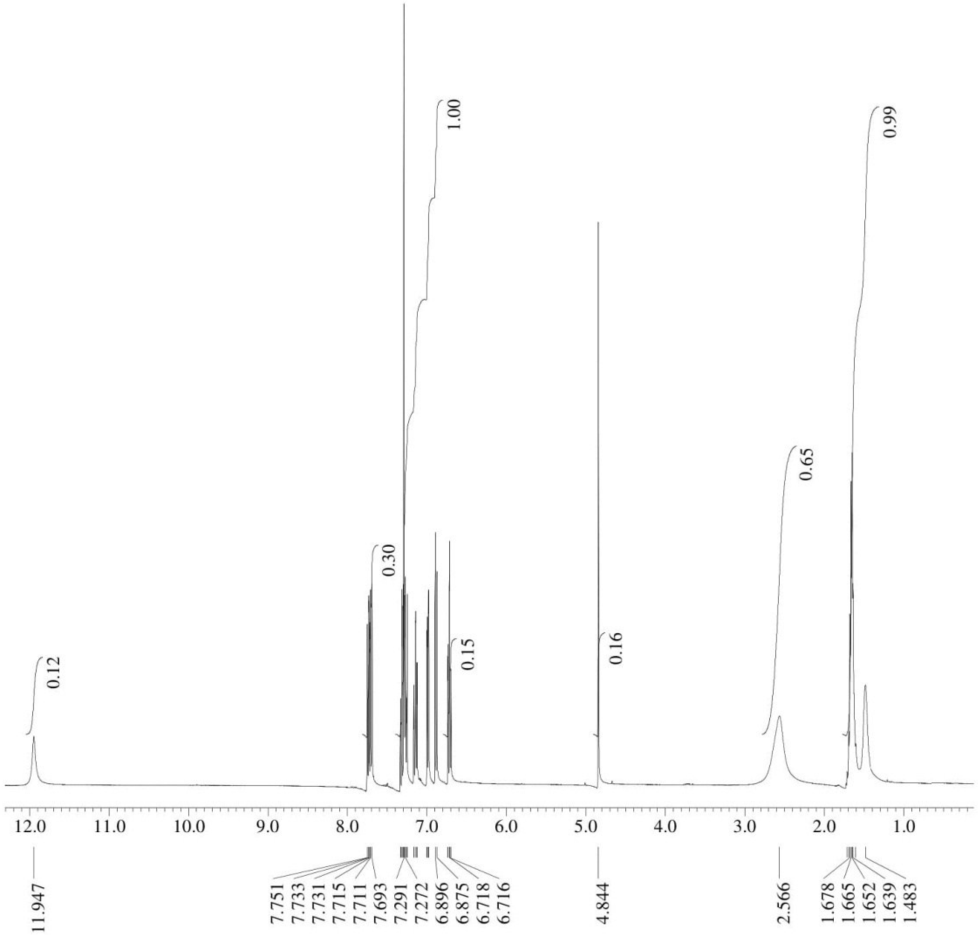

Figure 15: ^{1}H NMR spectrum of **(4k)**

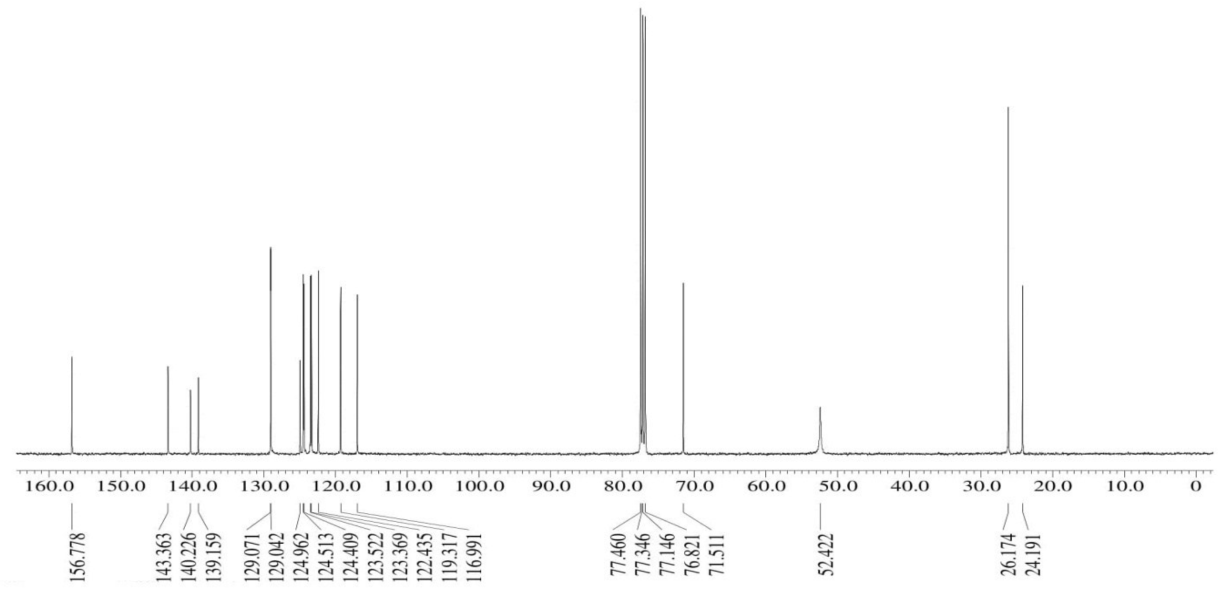

Figure 16: ^{13}C NMR spectrum of **(4k)**

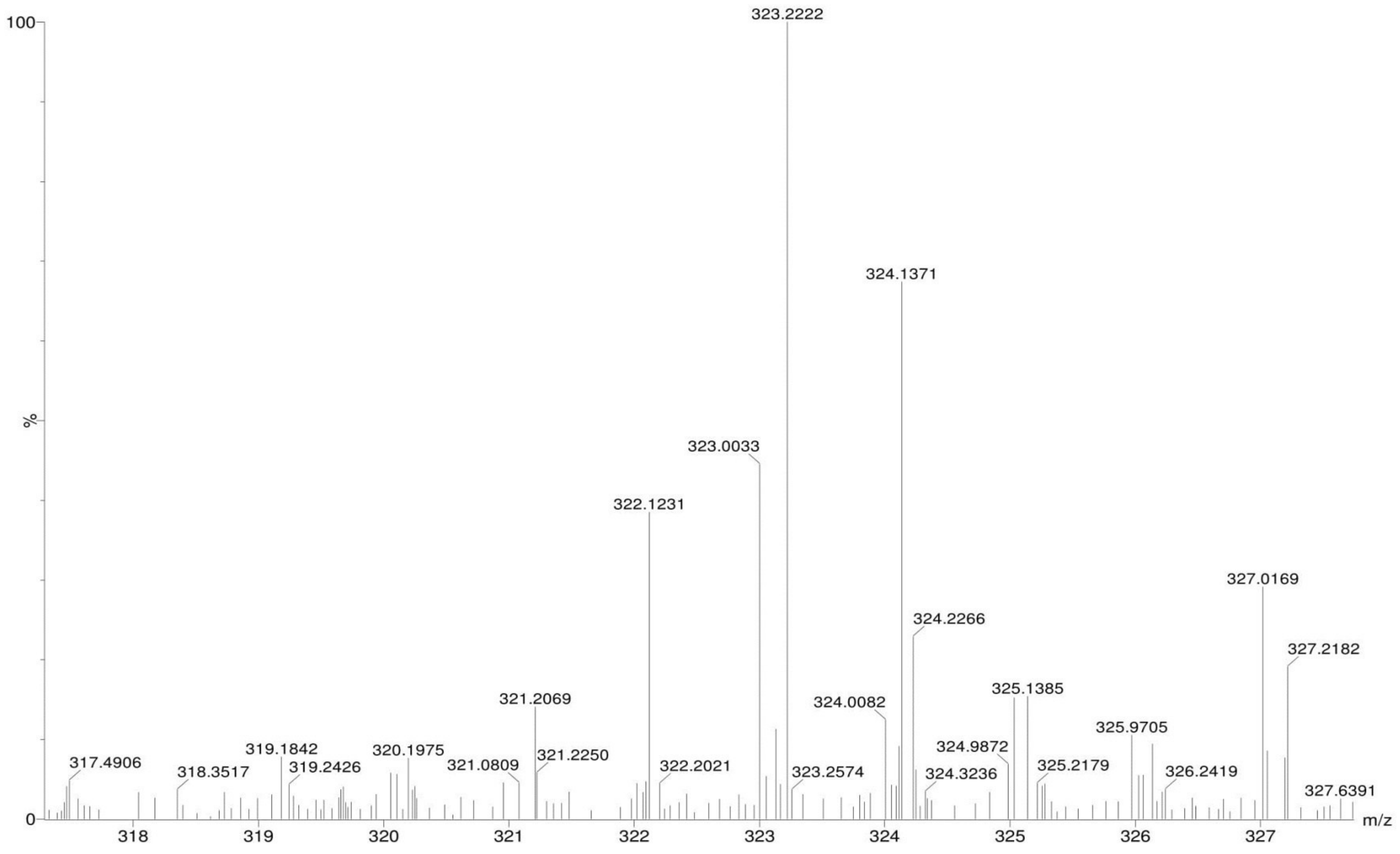

Figure 17: Mass spectrum of **(4k)**

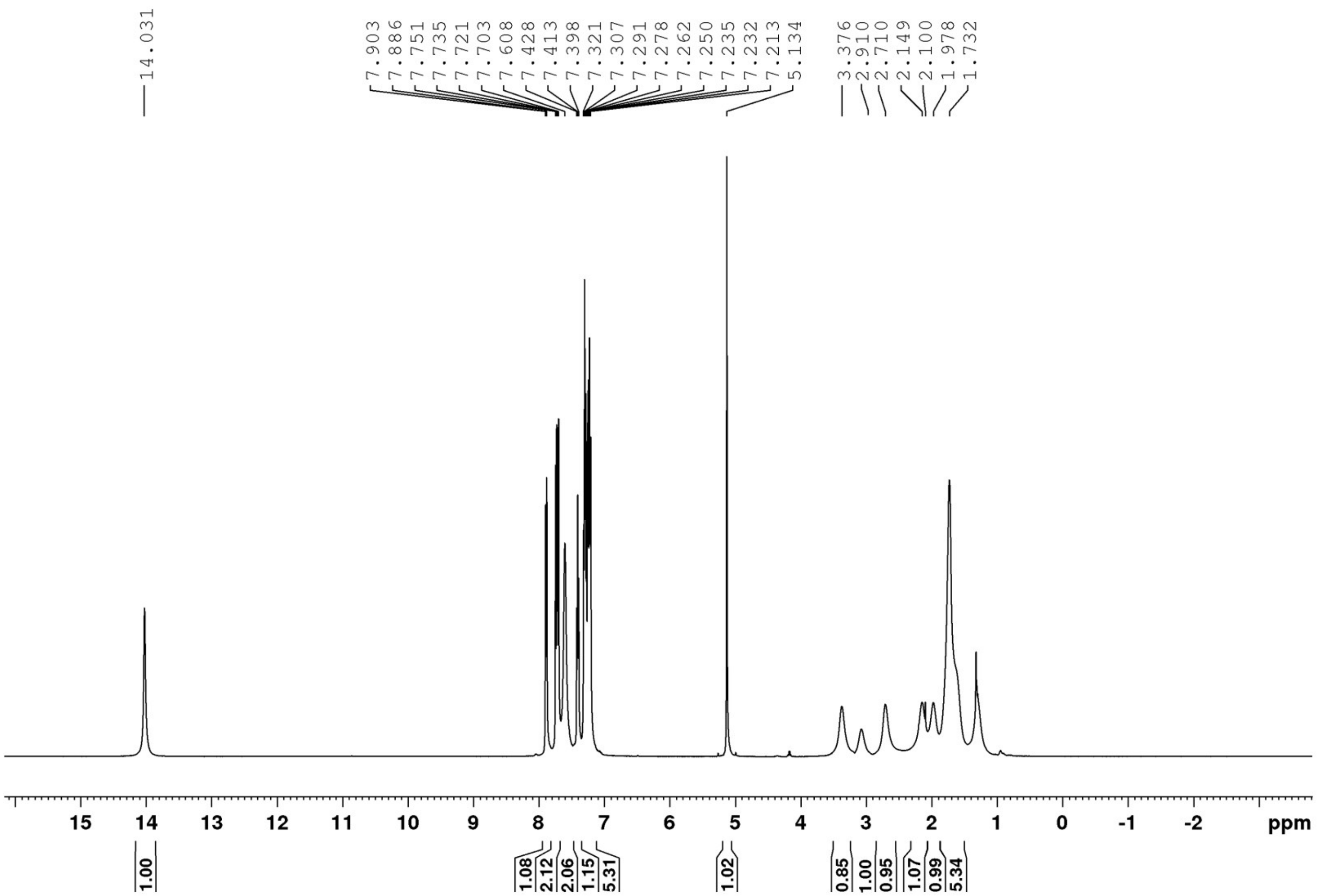

Figure 18: ^{1}H NMR spectrum of **(4o)**

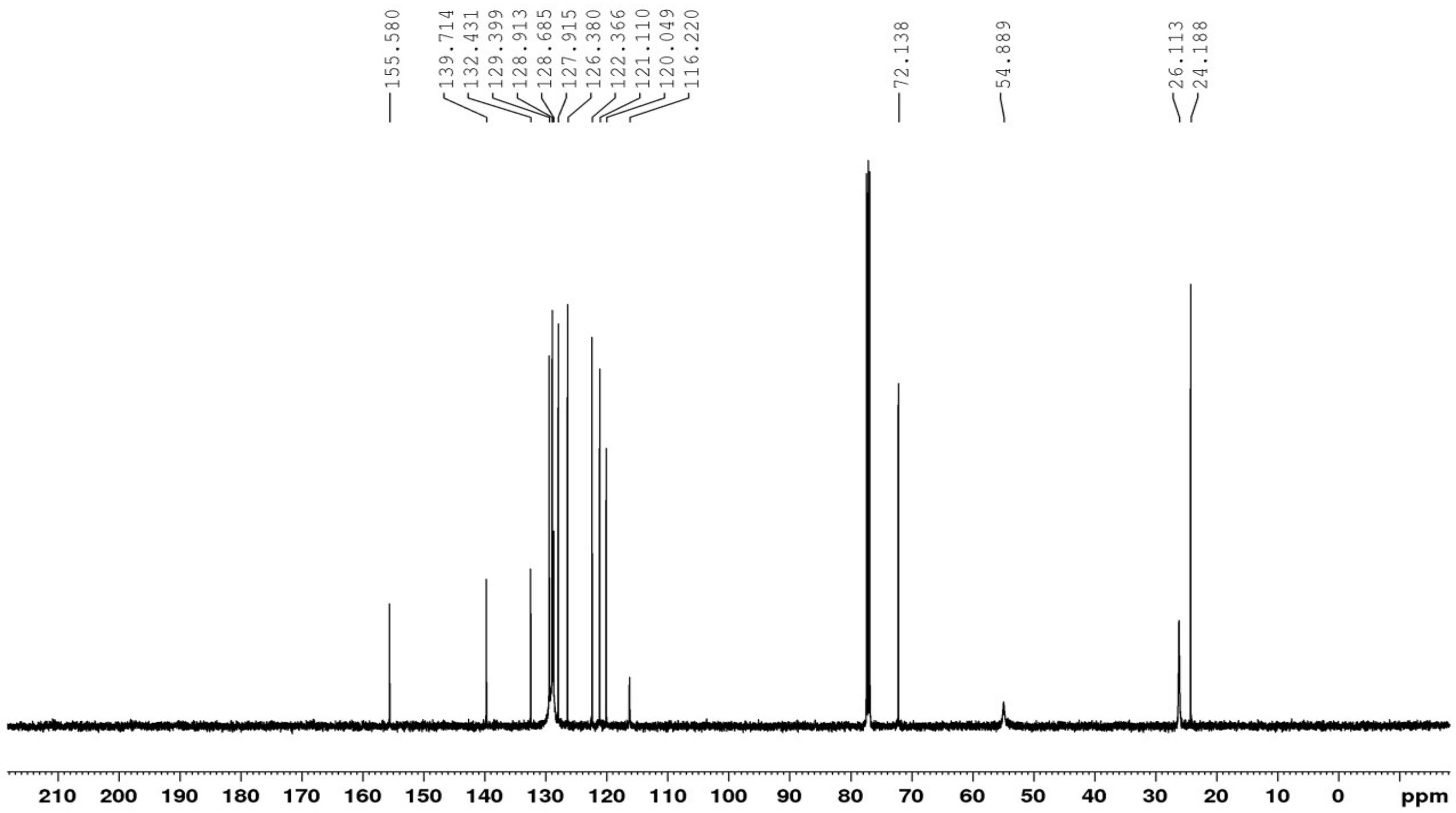

Figure 19: ^{13}C NMR spectrum of **(4o)**

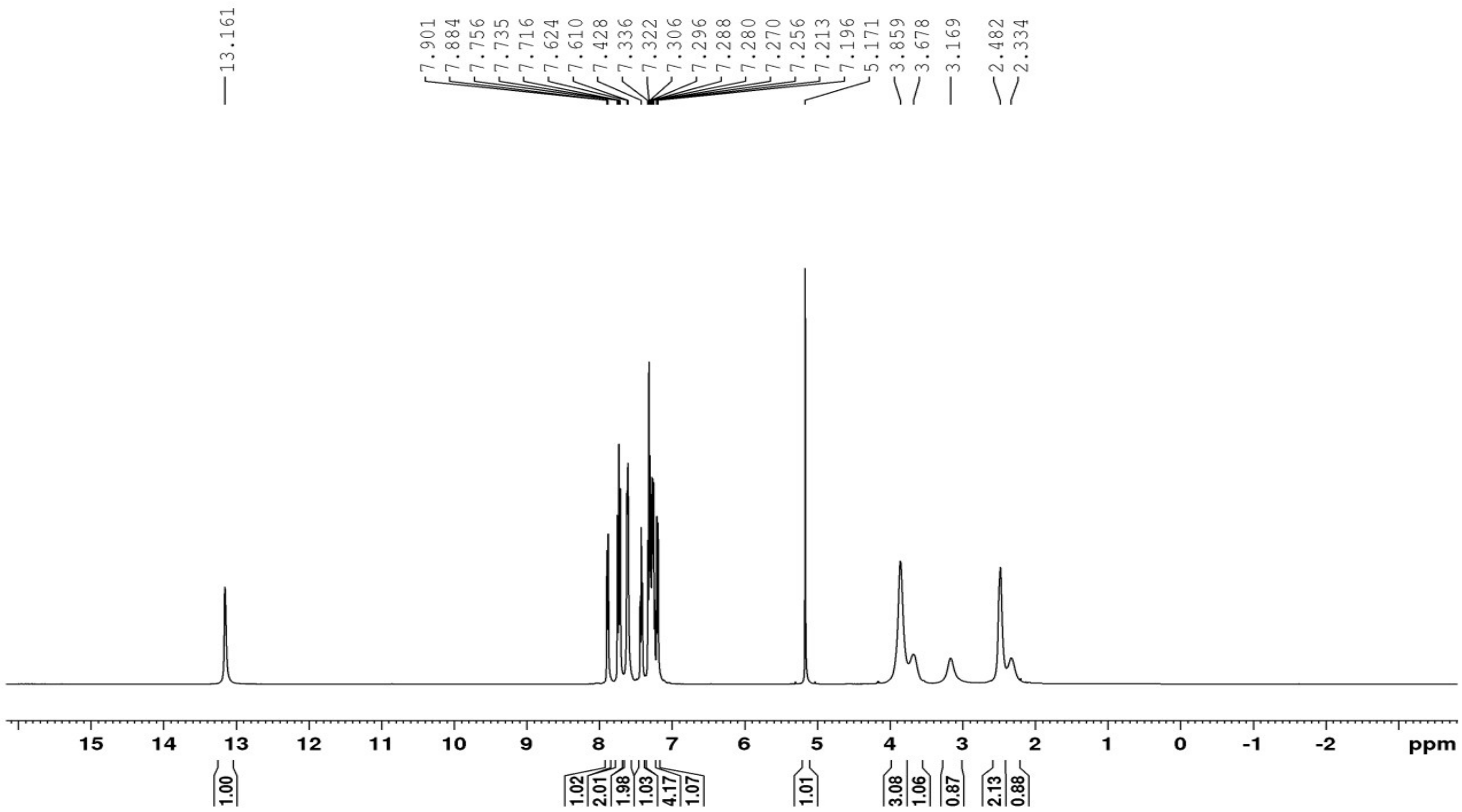

Figure 20: ^{1}H NMR spectrum of **(4p)**

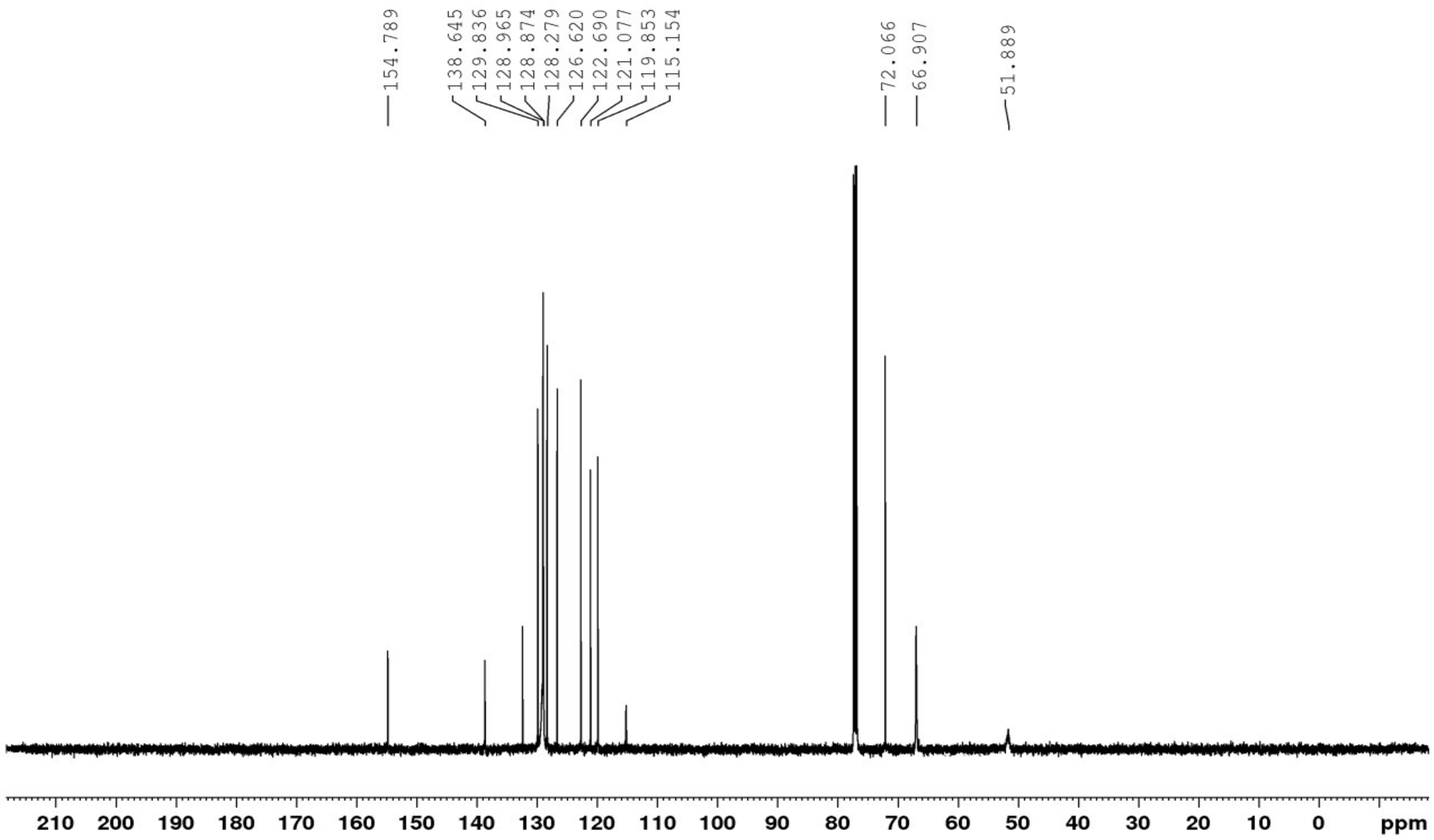

Figure 21: ^{13}C NMR spectrum of **(4p)**

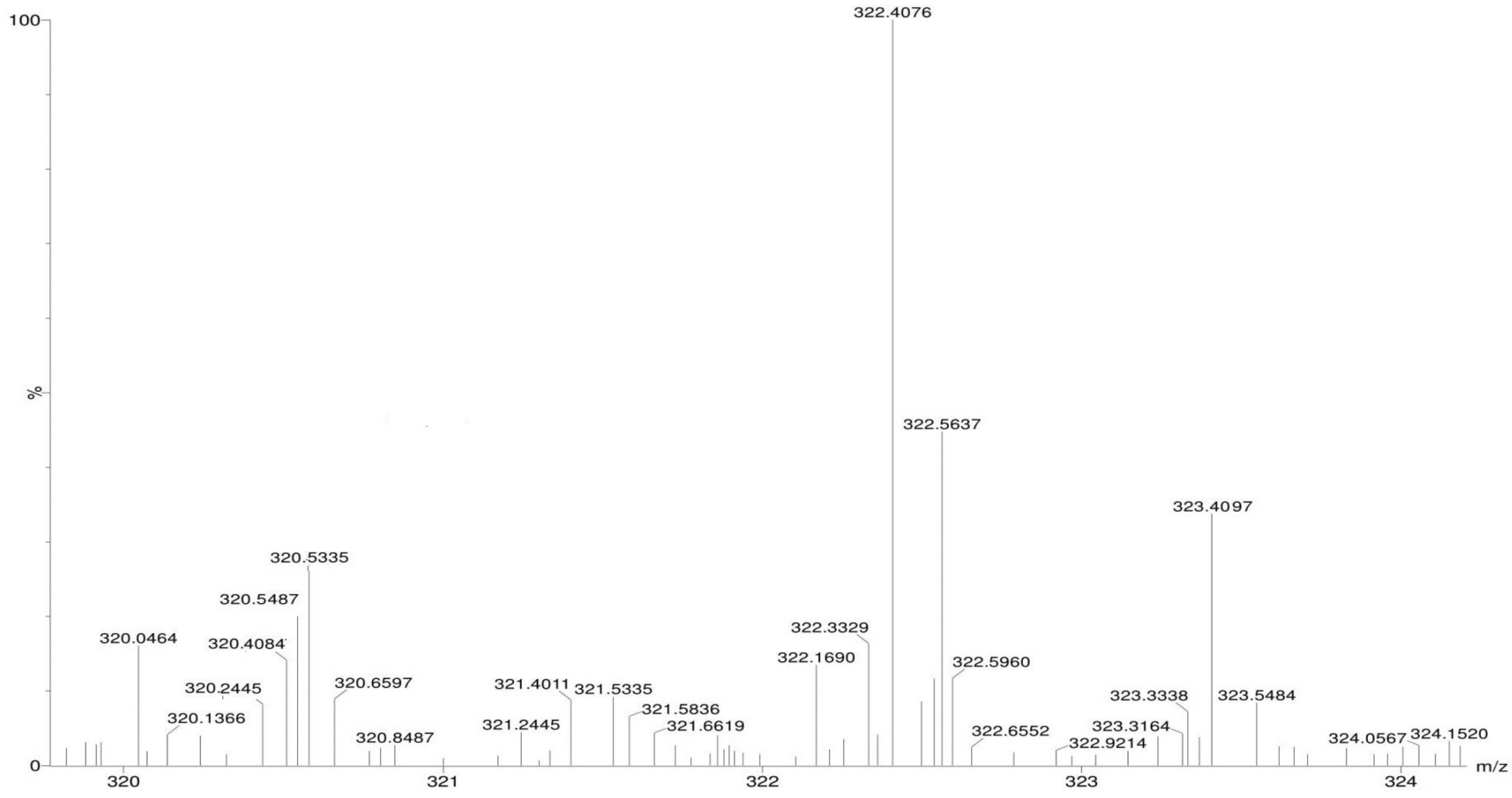

Figure 22: Mass spectrum of **(4p)**
